# Zebrafish as a robust preclinical platform for screening plant-derived drugs with anticonvulsant properties—a review

**DOI:** 10.3389/fnmol.2023.1221665

**Published:** 2023-08-28

**Authors:** Bartosz Knap, Dorota Nieoczym, Uday Kundap, Kamila Kusio-Targonska, Wirginia Kukula-Koch, Waldemar A. Turski, Kinga Gawel

**Affiliations:** ^1^Department of Experimental and Clinical Pharmacology, Medical University of Lublin, Lublin, Poland; ^2^Department of Animal Physiology and Pharmacology, Institute of Biological Sciences, Maria Curie-Skłodowska University, Lublin, Poland; ^3^Canada East Spine Center, Saint John Regional Hospital, Horizon Health Center, Saint John, NB, Canada; ^4^Department of Pharmacognosy with Medicinal Plants Garden, Medical University, Lublin, Poland

**Keywords:** zebrafish, mice, plant, plant-derived drugs, convulsion, epilepsy, screening, methodology

## Abstract

Traditionally, selected plant sources have been explored for medicines to treat convulsions. This continues today, especially in countries with low-income rates and poor medical systems. However, in the low-income countries, plant extracts and isolated drugs are in high demand due to their good safety profiles. Preclinical studies on animal models of seizures/epilepsy have revealed the anticonvulsant and/or antiepileptogenic properties of, at least some, herb preparations or plant metabolites. Still, there is a significant number of plants known in traditional medicine that exert anticonvulsant activity but have not been evaluated on animal models. Zebrafish is recognized as a suitable *in vivo* model of epilepsy research and is increasingly used as a screening platform. In this review, the results of selected preclinical studies are summarized to provide credible information for the future development of effective screening methods for plant-derived antiseizure/antiepileptic therapeutics using zebrafish models. We compared zebrafish *vs.* rodent data to show the translational value of the former in epilepsy research. We also surveyed caveats in methodology. Finally, we proposed a pipeline for screening new anticonvulsant plant-derived drugs in zebrafish (“from tank to bedside and back again”).

## 1. Introduction

### 1.1. Epilepsy

Epilepsy is a common neurological disease characterized by the occurrence of seizures caused by uncontrolled excessive discharges of neurons in the brain. The diagnosis of epilepsy requires two unprovoked or reflex seizure incidents at least 24 h apart. Epilepsy is also diagnosed in patients who have experienced only one seizure (unprovoked or reflex) if there is at least 60% likelihood of further seizure attacks to occur within the next 10 years, as well as in those patients who have been diagnosed with an epilepsy syndrome (Fisher et al., 2014). Epilepsy affects about 50 million people worldwide and remains an important problem for current medicine due to its varied etiology, complex clinical profile, and high morbidity and mortality rates (Duncan et al., 2006; World Health Organization, 2019). The disease can develop and manifest itself in people of all ages, from newborns to the elderly, although it is most commonly identified in children under the age of 10 and in adults over the age of 85 (Beghi, 2020). Every year, 61.4 per 100,000 people in the world develop epilepsy, and the incidence rate is significantly higher in low- and middle-income countries than in high-income countries, i.e., 139 *vs.* 48.9 per 100,000 people/year (World Health Organization, 2019; Beghi, 2020). One of the most important problems of seizure attacks is the increased risk of premature death, which in people with epilepsy is, on average, two or three times higher than in the general population (Trinka et al., 2023). There are several reasons for higher mortality, starting with sudden unexpected death in epilepsy or *status epilepticus*, ending with trauma, pneumonia, or suicide (Thurman et al., 2017). The standardized mortality rate ranges from 1.6 to 3.0 in high-income countries, while in low- and middle-income countries it reaches 19.8 (Thurman et al., 2017; Beghi, 2020; Trinka et al., 2023).

The first mentions of epilepsy and its treatment strategies were made at least 4,000 years ago, in Mesopotamia. Over the centuries, the understanding of epilepsy and the approach to its treatment have changed—from ineffective treatments of epilepsy involving skull trephination or bloodletting in ancient times, through a more religious approach in the Middle Ages, with epilepsy viewed as a sign of evil influence or occultism, to a modern approach due to the rapid development of medical sciences (Kaculini et al., 2021).

Epilepsy is not a homogenous disease. Its types can differ in terms of etiology and symptoms, as well as treatment response (Duncan et al., 2006; Scheffer et al., 2017). Pharmacotherapy is the basic treatment for epilepsy, and provides satisfactory seizure control and substantial improvements in the patient’s quality of life in most cases. The currently available antiseizure medications (ASMs) act through several main mechanisms, i.e., (1) the modulation of some voltage-gated ion channels, i.e., sodium (phenytoin, primidone, and eslicarbazepine), calcium (ethosuximide, pregabalin), and potassium (retigabine); (2) the potentiation of γ-aminobutyric acid (GABA) inhibitory neurotransmission (benzodiazepines, valproic acid, tiagabine, and vigabatrin); (3) the restriction of excessive glutamatergic neurotransmission (perampanel); and (4) neurotransmitters, i.e., glutamate and GABA release modulation (levetiracetam, brivaracetam, gabapentin, and pregabalin); (Sills and Rogawski, 2020; Łukasiuk and Lasoń, 2023). Nevertheless, approximately 30% of all patients with epilepsy continue to experience seizures despite using available ASMs as mono- or polytherapy (Guerreiro, 2016; Hakami, 2021). In addition, ASMs, especially first-generation drugs, cause some adverse effects, among which the most serious and troublesome are depression, anxiety, mood changes, cognitive dysfunction, sleep disturbances, and motor impairments. In many cases, such adverse effects are so disruptive to patient functioning that they decide to discontinue therapy—even if it effectively controls seizures (Abou-Khalil, 2016; Guerreiro, 2016; Hakami, 2021). An additional disadvantage of ASMs is that they only treat the symptoms of epilepsy, but do not cure the cause(s) of the disease—meaning that they do not prevent or inhibit epileptogenesis (the process that changes a healthy brain into the epileptic); (French and Perucca, 2020). All these problems confront modern medicine and compel the undertaking of the ambitious task to look for new therapeutics that will not only reduce seizure severity and/or frequency (and have less adverse effects), but will primarily be able to prevent epileptogenesis.

In the past decade, as the knowledge of the genetic background of the diseases and gene manipulation techniques has increased, many researchers have been developing genetically modified disease models to study drug response. Pharmacogenetic studies show how drug response is affected by genotype (Fontana et al., 2018). The rapid disease-gene discoveries have resulted in enormous development and understanding in the field of genetic epilepsy (Myers et al., 2019). Human genome sequencing has become much easier since 2003, which was when human gene sequencing was fully explored, and the gene panels, exomes and genomes available have led to higher diagnostic rates and a better understanding of the disease processes (Gonzaga-Jauregui et al., 2012). The development of recent technology has supported genetic discovery in epilepsy and the molecular mechanisms of many epileptic diseases have been increasingly understood, eventually providing targets for a precision medicine approach to epilepsy treatment (Kearney et al., 2019).

### 1.2. Plants as a source of anticonvulsants

Plants with anticonvulsant properties have been known for centuries and are also currently used in the treatment of epilepsy/seizures in many cultures, especially in low-income countries with poor medical systems (Aghdash, 2021; Birhan, 2022; Faheem et al., 2022). Anticonvulsant remedies of plant origin are traditionally administered in the form of infusions, decoctions or powders; however, numerous species are also ingested as regular food. Limited access to modern synthetic drugs makes herbal preparations the main, or even the only, treatment for the disease in the developing countries (Liu et al., 2017). Developed countries medicine is more skeptical to the use of herbal preparations. Nevertheless, there are several plant-derived drugs known to effectively treat patients suffering from epilepsy. A good example is valproic acid. This is a derivative of valeric acid—a compound naturally present in the roots of *Valeriana officinalis*. Valproic acid was first synthesized in 1882 by Beverly Burton, an American chemist, and it was initially used as a pharmacologically inert solvent in medical research. In 1962, scientists found that proconvulsant substances dissolved in valproic acid did not induce seizures. This finding led to approving valproic acid as an anticonvulsant drug in 1972, in France. Nowadays, this is one of the most commonly used drugs in diverse forms of epilepsy (Tomson et al., 2016). Another example of an ASM with strict plant origin is cannabidiol (CBD)—a cannabinoid naturally present in *Cannabis* spp. CBD was found to effectively inhibit seizures in patients with Dravet or Lennox–Gastaut syndromes—serious developmental and epileptic encephalopathies that are generally resistant to treatment (García-Peñas et al., 2021). The U.S. Food and Drug Administration (FDA) and the European Medicines Agency (EMA) have approved CBD to treat these syndromes even in 1- and 2-year-old children (Devinsky et al., 2017, 2018). Moreover, clinical trials of huperzine A, a compound isolated from the Chinese plant *Huperzia serrata*, are currently being conducted among adult patients with pharmacoresistant epilepsy (Clinicaltrials.gov/ Identifier: NCT03474770, NCT05518578).

Preclinical studies on animal models of seizures and epilepsy have revealed the anticonvulsant and/or antiepileptogenic properties of numerous herb preparations and plant-derived drugs. Indeed, some of the studied plants have been used in traditional ethnomedicine (Zhu et al., 2014; Kwon et al., 2019; Bertoncello and Bonan, 2021; Sharifi-Rad et al., 2021). However, there is a lack of robust results from preclinical studies that could objectively confirm or disaffirm their use as potential ASMs (Liu et al., 2017).

### 1.3. Zebrafish

Zebrafish (*Danio rerio*) is a small striped minnow natural to the freshwaters of the Indian subcontinent. Since their first scientific use in the 1960s, the popularity of zebrafish in biomedical sciences has been increasing at a rapid pace (Stewart et al., 2014; Crouzier et al., 2021; Al-Hamaly et al., 2023; Chang et al., 2023; Liu, 2023; Tesoriero et al., 2023). Zebrafish is a very popular organism for high-throughput screening studies (Baxendale et al., 2012; Baraban et al., 2013; Asad et al., 2023). Larval zebrafish have also gained popularity as an animal model for the study of the molecular mechanisms of brain diseases at early developmental stages and for testing potential therapeutic drugs (Grone and Baraban, 2015; Kundap et al., 2017).

There is definitely a long list of advantages of zebrafish use for biomedical studies, especially for epilepsy research (Stewart et al., 2014, 2015; Bertoncello and Bonan, 2021; Yaksi et al., 2021; D'Amora et al., 2023). Briefly, there is a high homology of zebrafish and human genomes, with at least one zebrafish orthologue existing for 70% of all human-disease related genes (Howe et al., 2013). There is also a high physiological similarity between zebrafish and humans in terms of molecular pathways, neurotransmitters, and receptors (Kalueff et al., 2014; Stewart et al., 2015; Jurisch-Yaksi et al., 2020). All this makes zebrafish an excellent model for genetic studies and for the creation of genetically altered animal models.

The zebrafish brain consists of the forebrain, the midbrain, the hindbrain, and the spinal cord, and brain structures like the cerebellum or the thalamus are homologous to human structures (Guo et al., 2012; Wullimann et al., 2012). There are, however, some differences between zebrafish and mammal brains, e.g., zebrafish lack the neocortex, and the telencephalon, in contrast to mammals, develops through eversion and not through invagination (Wullimann et al., 2012). The blood–brain barrier in zebrafish, however, is complex and similar to that of mammals. It starts to develop at 3 days post-fertilization (dpf) becoming mature at 10 dpf (Jeong et al., 2008; Fleming et al., 2013; Quiñonez-Silvero et al., 2020). This has to be taken into account when planning experiments using larvae at this stage of development.

**Figure 1:**
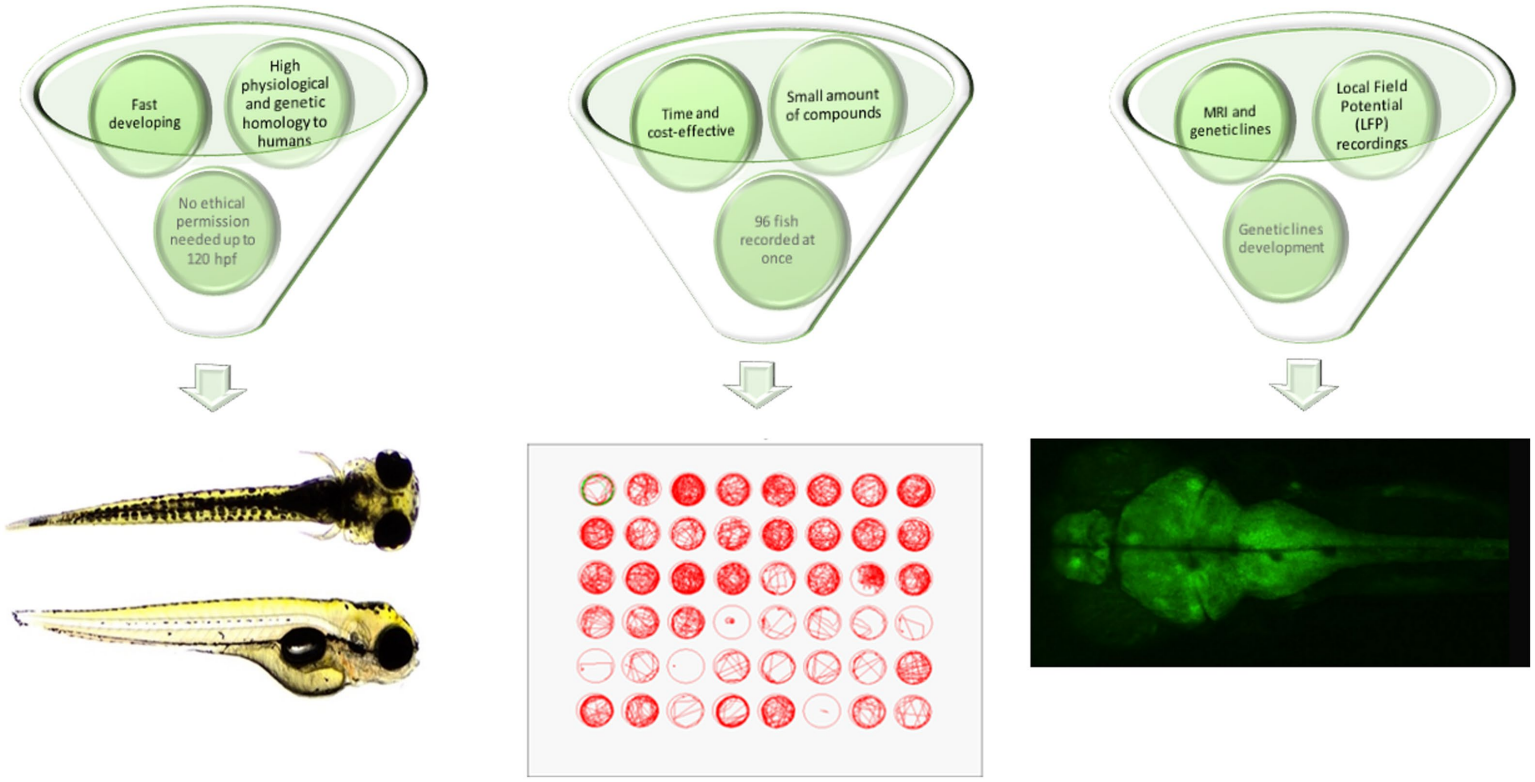
Advantages of larval zebrafish for high-throughput screening of plant-derived drugs with anticonvulsant properties—a summary. hpf, hours post fertilization; MRI, magnetic resonance imaging.

From the practical point of view, zebrafish are quick and easy to breed, with low maintenance costs compared to rodents. The small size of zebrafish embryos and larvae substantially decreases the amounts of substances, drugs or extracts used during screening assays. In addition, the minute body size of the larvae allows the concomitant screening of up to 96 larval zebrafish within one time frame, which substantially speeds up the entire process of screening and makes it more efficient and less expensive. According to current European Union legislation, testing the larval zebrafish up to 120 h post-fertilization (hpf), i.e., the stage of being capable of independent feeding, does not require any ethical permission (Figure 1).

Zebrafish have been frequently employed as model organisms in epilepsy research (Gawel et al., 2020b; Crouzier et al., 2021; Yaksi et al., 2021). Currently, the best validated model of convulsions in larval zebrafish consists of animal exposure to pentylenetetrazole (PTZ; Table 1). Other proconvulsant agents (e.g., picrotoxin, kainic acid, or pilocarpine) are less validated and utilized rarely, but, similar to PTZ, they are still used more frequently in larvae than in adult zebrafish (Gawel et al., 2020b). Therefore, in most but not all cases, researchers employ the PTZ-induced seizure model as their first-choice screening platform (although it is worth noting that *scn1Lab* zebrafish mutants were utilized to identify anticonvulsant properties of clemizole; Baraban et al., 2013). Interestingly, PTZ added to the medium induces abrupt hyperlocomotion, both in larvae and adult zebrafish. One may assume that hyperlocomotion reflects clonic–tonic-like seizures in humans (Baraban et al., 2005; Wong et al., 2010; Afrikanova et al., 2013). Furthermore, pretreatment with anticonvulsant compounds decreases the speed and/or distance traveled by PTZ-exposed zebrafish (Baraban et al., 2005; Afrikanova et al., 2013). This approach has been widely adopted in the scientific community (Baxendale et al., 2012; Orellana-Paucar et al., 2012; Buenafe et al., 2013; Gawel et al., 2021).

**Table 1:** A summary of the zebrafish models of seizures/epilepsy described in this review.

Intervention	Primary molecular mechanism	Putative type of seizures/epilepsy	Reference (zebrafish model)
PTZ-induced seizures	GABAergic neurotransmission decline	Generalized clonic–tonic seizures	Afrikanova et al. (2013)
EKP-induced seizures	Glutamic acid decarboxylase (GAD) inhibition	Pharmacoresistant epilepsy	Zhang Y. et al. (2017)
*gabra1^−/−^* zebrafish mutants	GABA_A_ receptor alpha1 subunit loss of function	Autosomal dominant juvenile myoclonic epilepsy	Samarut et al. (2018)
*gabrg2^−/−^* zebrafish mutants	GABA_A_ receptor gamma 2 subunit loss of function	Absence epilepsy and febrile seizures	Liao et al. (2019)
*scn1Lab^−/−^* zebrafish mutants	Voltage-gated sodium channel subunit Na_V_1.1 loss of function	Dravet syndrome (severe myoclonic epilepsy of infancy)	Baraban et al. (2013); Thornton et al. (2020)

EKP - ethyl ketopentenoate; GABA- γ-aminobutyric acid; PTZ- pentylenetetrazole.

Bertoncello and Bonan (2021) elaborated data concerning various plants and/or their isolated constituents that were investigated for antiseizure activity using zebrafish as a screening platform. In their review, the authors focused mostly on discovered drugs. Here, particular emphasis was placed on assessing the anticonvulsant effect(s) of the plant extract(s) or isolated plant-derived drug(s), estimated *via* different models of convulsions/seizures, which were recorded using multiple testing methods. In our study, we selected and evaluated only the most comprehensive papers in this field. That which contained too many gaps (e.g., lack of critical details of the methodology used, insufficient description of plant extraction, and invalid description of the locomotor activity assay used) were not included. This allowed us to identify and survey caveats, e.g., in a methodology which should be considered in future experiments to move the field further on. Moreover, we gathered and compared zebrafish and rodent data to show the translational value of zebrafish in epilepsy research. Finally, we proposed a pipeline for screening new anticonvulsant drugs of plant origin in zebrafish (“from tank to bedside and back again”).

## 2. Plant extracts and/or active constituents with anticonvulsant activity

### 2.1. *Berberis* spp.

*Berberis* spp. (Berberidaceae; barberry) are found mainly in the temperate zone of the northern hemisphere, and they are used both as a food flavor and in traditional medicine to treat insomnia, liver, and bronchial diseases, as well as afflictions of the urinary and gastrointestinal systems (Abdykerimova et al., 2020). Extracts from *Berberis* spp. have been found to display antimicrobial, anti-inflammatory and antinociceptive properties. The anticonvulsant properties of *Berberis L. vulgaris* and *Berberis integerrima* Bunge. extracts were previously demonstrated in rodent models of seizures (Hosseinzadeh et al., 2013; Khosravi Dehaghi et al., 2017; Supplementary Table 1), while PTZ-induced seizure assay in the zebrafish larvae was employed to evaluate the anticonvulsant potential of the *Berberis sibirica* root extract (Gawel et al., 2020a; Table 2).

**Table 2:** Anticonvulsant effects of plant extracts or plant-derived drugs in different seizure or epilepsy models in zebrafish.

**Plant**	**Active extract/ plant-derived drug** (concentration, time of incubation)	**Model of convulsions/seizures/epilepsy and noticed effect**	**LFP recordings**	**Other findings/conclusions**	**Reference**
Berberis sibirica	Berberine (25-75 μM, 2 days)	PTZ-induced seizure model in dark condition – ↓ total distance and mean velocity; PTZ-induced seizure model under dark/light shift condition (photosensitive seizures) – ↓ total distance and mean velocity		↓ *c-fos* gene expression and ↑ *stx1b* gene expression; Anticonvulsant effect of berberine might be at least partially mediated by STX1B.	(Zheng et al., 2018)
	Berberine (100 μM, 2 days)	PTZ-induced seizure model – ↓ total distance and average speed	↓ frequency of neuronal discharges	↓ *c-fos* gene expression; ↓ PTZ-induced neutrophil and macrophages migration into the brain; ↓ *TNFα*, *IL-1β* and *IL-6* mRNA expression. Anticonvulsant effect of berberine might result from its anti-inflammatory properties.	(Zhang et al., 2020)
	Palmatine (75-450 μM, 20 h); Berberine (100-200 μM, 20 h)	PTZ-induced seizure assay – ↓ hyperlocomotion (palmatine and berberine)	↓ number and mean duration of events in the optic tectum (palmatine and berberine)	Palmatine – ↓ *c-fos* and *bdnf* mRNA expression; Combination of palmatine and berberine at their ED_16_ doses revealed hyperadditive activity in the PTZ-induced seizure test. Combination of palmatine and berberine in a ratio of 1:2.17 (as in the pure methanolic extract from *Berberis sibrica* roots) showed the same anticonvulsant activity in the PTZ test as the pure extract.	(Gawel et al., 2020a)
	Naringenin (12.5-50 μM, 1h); Kaempferol (25 μM, 1h); Naringenin 7-*O*-methyl ether (12.5-50 μM, 1h); Naringenin 4',7-dimethyl ether (12.5-50 μM, 1h); Kaempferide (4'-*O*-methyl kaempferol) (25 μM, 1h)	PTZ-induced seizure test – ↓ hyperlocomotion – kaempferol, naringenin 7-*O*-methyl ether, naringenin 4',7-dimethyl ether, kaempferide (4'-*O*-methyl kaempferol); the most efficient were naringenin deivatives, while kampferol and kaempferide had limited activity	Kaempferol, naringenin 7-*O*-methyl ether, naringenin 4',7-dimethyl ether – ↓ percentage of larvae with epileptiform activity. Naringenin 7-*O*-methyl ether, naringenin 4',7-dimethyl ether – ↓ number and cumulative duration of PTZ-induced epileptiform-like events	Naringenin 7-*O*-methyl ether increased locomotor activity in the control zebrafish larvae. Both naringenin and naringenin 7-*O*-methyl ether alone increased percentage of zebrafish larvae with epileptiform activity.	(Copmans et al., 2018)
*Cannabis sativa*	THC (1.5-3 μM, 1 h); CBD (1.5-2.5 μM, 1 h)	PTZ-induced seizure test – ↓ total distance and fast activity		Combination of THC and CBD at a ratio 1:1 had synergistic effect both in the PTZ test as well as in the genetic model.	(Samarut et al., 2019)
	THC (6-7 μM, 1 h); CBD (5-7 μM, 1 h)	GABRA1 deficiency (*gabra1^-/-^*) zebrafish line – ↓ maximum acceleration upon light			
	THC (1-4 μM, 24 h); CBD (0.3-1 μM, 24 h); CBN (0.3-1 μM, 24 h); LN (0.3-4 μM, 24 h)	PTZ-induced seizure model – THC and CBD – ↓ total distance; *scn1Lab^–/–^* zebrafish line – THC, CBD, CBN and LN – ↓ total distance			(Thornton et al., 2020)
*Curcuma longa*	Methanolic exctract (3.1-12.5 μg/ml, 1 h); Curcuminoids (2.5-10 μg/ml, 1 h); Turmeric oil (2.5-10 μg/ml, 1 h); Ar-turmerone (11-46 μM, 1 h); α,β-turmerone (5-23 μM, 1 h); α-atlantone (11-46 μM, 1h)	PTZ-induced seizure model – the studied extract and all compounds – ↓ total movements	Turmeric oil – ↓ number and duration of ictal-like discharges as well as cumulative duration of all forms of epileptiform-like discharges in the PTZ-exposed zebrafish larvae	Curcuminoids, turmeric oil, α,β-turmerone, ar-turmerone and α-atlantone alone slight increased locomotor activity of the zebrafish larvae compared to the vehicle-treated controls.	(Orellana-Paucar et al., 2012)
	Curcumin (larvae form – 1μM, adult – 0.5 mg/kg, 30 min); Micronized curcumin (larvae form – 1μM, adult – 0.5 mg/kg, 30 min)	PTZ-induced seizure model – micronized curcumin – ↓ seizure occurrence and ↑ latency to seizures stage I and II; curcumin and micronized curcumin – ↑ latency to seizures stage III			(Bertoncello et al., 2018)
	Curcumin (5 μM, 2 h); 68 curcumin derivatives (5 μM, 2 h)	PTZ-induced seizure model – curcumin – ↓ total distance and maximum acceleration; 15 curcumin derivatives – ↓ maximum acceleration; *gabra1^-/-^* zebrafish line – curcumin (12.5 μM) – ↓ maximal acceleration; 3 curcumin derivatives (i.e., A 20, 4-Cl CA, MS 57) – ↓ maximal acceleration; *gabrg2^-/-^* zebrafish line – curcumin (12.5 μM) – ↓ maximal acceleration; 6 curcumin derivatives (i.e., CA, CA 18, CA 20, Ca 74, CA 80, MS 55) – ↓ maximal acceleration		Fluorescence detection of neuronal firing in transgenic reporter line (i.e., *Tg[neurod:GCaMP6f]*) of zebrafish – *gabra1^-/-^* zebrafish line – none of the tested compounds were effective; *gabrg2^-/-^* zebrafish line – 2 curcumin derivatives (i.e., CA 80 and ca 74) – ↓ neuronal activity in the optic tectum. Only 1 curcumin derivative was active in all behavioral seizure assays. Curcumin at concentration of 15 μM was lethal for both genetically modified zebrafish lines.	(Choo et al., 2021)
*Garcinia oligantha*	Oliganthin H (6.2-25 μM, 6 h); Oliganthin I (3.1-12.5 μM, 6 h); Oliganthin N (0.2-0.7 μM, 6 h)	PTZ-induced seizure test – oliganthin H – ↓ total distance travelled, velocity, seizure activity and duration, number of burst; oliganthin N – ↓ total distance travelled; oliganthin I – ineffective		Oliganthin H – ↓ *npas4a*, *c-fos*, *pyya, bdnf, gabra1*, *gad1*, *glsa*, *glula* mRNA expression. *In silico* study revealed that oliganthin H has strong binding potency to the GABA_A_ receptor and this complex is characterised by good stability.	(Gong et al., 2020)
*Indigofera arrecta*	Methanolic extract from leaves; Indirubin (30-300 μM, 18 h)	PTZ-induced seizure test – the methanolic extract and indirubin – ↓ hyperlocomotion	Indirubin – ↓ number of interictal and ictal-like spikes, total duration of epileptiform activity	Anticonvulsant effect of indirubin might at least partially result from inhibition of glycogen synthase kinase (GSK)-3.	(Aourz et al., 2019)
*Magnolia officinalis*	Ethyl acetate extracts from barks (0.5-4 μg/ml, 3 h); Magnolol (0.5-4 μM, 4 h)	PTZ-induced seizure test – ethyl acetate extract – ↓ hyperlocomotion and ↑ latency to seizure stage III; magnolol – ↓ hyperlocomotion; honokiol – slight ↓ hyperlocomotion			(Moradi-Afrapoli et al., 2017)
	Ethanol extract from barks (1.57-6.25 μg/ml, 2 h); Acetone extract from barks (3.13-12.5 μg/ml, 2 h); Magnolol (3.13-12.5 μg/ml, 2 h); Honokiol (1.57-6.25 μg/ml, 2 h)	PTZ-induced seizure test – both tested extracts – ↓ seizure-like movements; magnolol and honokiol – ↓ seizure-like movements; EKP-induced seizures – both tested extracts – ↓ seizure-like movements; magnolol and honokiol – ↓ seizure-like movements	Magnolol and honokiol – ↓ frequency and cumulative duration of epileptiform-like PTZ- and EKP-induced events and ↓ power spectral density of LFPs in the PTZ- or EKP-treated zebrafish larvae	Although magnolol and honokiol increased locomotor activity of control zebrafish larvae, they did not cause any significant changes in the brain activity that was assessed in the electrophysiological analysis.	(Li et al., 2020)
	Pterostilbene (10 μM, 2 h)	PTZ-induced seizure test – ↓ hyperlocomotion	↓ number, mean and cumulative duration of epileptiform-like events		(Nieoczym et al., 2019)
*Solanum torvum*	Aqueous extract from the aerial parts (50-200 μg/ml, 18 h); Methanol crude extract from the aerial parts (50-200 μg/ml, 18 h); Traditional water decoction from the aerial parts (50-200 μg/ml, 18 h); Torvoside J (35-140 μM, 18 h) Torvoside L (70-280 μM, 18 h); Torvoside K (70-280 μM, 18 h)	PTZ-induced seizure test – all tested extracts – ↓ seizure-like movements; torvoside J – ↓ seizure-like movements; torvoside L and torvoside K – slight ↓ seizure-like movements			(Challal et al., 2014)
*Valeriana officinalis*	Ethyl acetate extract from roots (6-12 μg/ml, 3 h); Valerenic acid (1.25-10 μM, 3 h)	PTZ-induced seizure test – the extract and valerenic acid – ↓ hyperlocomotion			(Moradi-Afrapoli et al., 2017)
*Zingiber purpureum*	Hexane extract from roots (10-30 μg/ml, 18 h); *Trans*-banglene (8-25 μM, 18 h); *Cis-*banglene (8-50 μM, 18 h)	PTZ-induced seizure test – the extract, *trans*-banglene and *cis-*banglene – ↓ hyperlocomotion		*Cis-*banglene revealed 4-8 more potent effect than topiramate in the PTZ seizure assay in the zebrafish larvae.	(Brillatz et al., 2020)
*Zingiber officinale*	Methanolic extract from roots (60 μg/ml, 24 h); 6-gingerol (12.5-37.5 μM, 24 h)	PTZ-induced seizure test – the extract and 6-gingerol – ↓ hyperlocomotion	6-gingerol – ↓ number and mean duration of epileptiform-like events in the PTZ-treated zebrafish larvae	Neurotransmitters’ concentration – 6-gingerol – ↓ glutamate level and glutamate/GABA ratio in the PTZ-treated zebrafish larvae; 6-gingerol – ↓ *grin2b* mRNA expression; 6-gingerol might act as an inhibitor for NMDA receptor and interact with the amino terminal domain, the glutamate-binding site, as well as within the ion channel of the NR2B-containing NMDA receptor.	(Gawel et al., 2021)

CBD, canabidiol; CBN, cannabinol; EKP, ethyl ketopentenoate; LFP, local field potential; LN, linalool; NMDA, N-methyl-D-aspartate; PTZ, pentylenetetrazole; THC, delta(9)-tetrahydrocannabinol.

Berberine and palmatine (*O*,*O*-dimethyldemethyleneberberine) are the main isoquinoline alkaloids present in *Berberis sibirica,* and they are responsible for its pharmacological properties. These compounds are also identified in other plants from the *Berberidaceae*, *Papaveraceae*, and *Ranunculaceae* families (Tarabasz and Kukula-Koch, 2020; Zhang et al., 2020). Plants containing berberine and palmatine have been exploited for centuries in traditional oriental medicine as a remedy for various ailments and diseases. Therapeutic effects of berberine include lowering body temperature, detoxification, analgesic, spasmolytic, and antihypertensive activity (Filli et al., 2020). Palmatine has been reported to be used as a remedy in jaundice- and liver-related diseases, hypertension, inflammation, dysentery, as well as in infections of the urinary, gastrointestinal and respiratory tracts (Tarabasz and Kukula-Koch, 2020).

The anticonvulsant activity of berberine was first evaluated in rodent models of seizures and epilepsy (Shanbhag et al., 1970; Bhutada et al., 2010; Gao et al., 2014; Mojarad and Roghani, 2014; Sadeghnia et al., 2017; Sedaghat et al., 2017; Supplementary Table 1), and then was investigated in larval zebrafish seizure tests (Zheng et al., 2018; Zhang et al., 2020; Table 2). Zheng et al. (2018) demonstrated the anticonvulsant action of berberine in two variants of PTZ-induced seizure tests in larval zebrafish, i.e., in tests conducted in the dark condition, as well as under dark/light shift condition (four cycles of 5-min dark and 10-s light periods). Light stimulation was applied to modulate photosensitive seizures that are at least partially mediated by syntaxin 1b (*stx1b*) deficiency, and in zebrafish larvae, alkaloid concentration-dependently limited the PTZ-induced hyperlocomotion both under dark and light stimulation conditions. The behavioral observation was correlated with the reduction of *c-fos* expression and the recovery of *stx1b* expression in the brain of the PTZ-treated zebrafish larvae. The possibility of quickly adopting genetic modification in zebrafish larvae allowed the identification of potential mechanisms responsive to the anticonvulsant action of berberine, and *in situ* hybridization was used to show changes in the *c-fos* and *stx1b* expression in their brains (Zheng et al., 2018).

Previous studies have revealed that the anticonvulsant effect of berberine in the zebrafish larvae PTZ-induced seizure assay might also result from its anti-inflammatory properties. In addition to reducing the seizure-like hyperlocomotion and *c-fos* expression, berberine decreased the number of spikes in local field potential (LFP) recordings. Inflammatory reactions associated with the seizure activity, i.e., macrophages and neutrophils recruitment, and increased expression of some inflammatory markers (i.e., tumor necrosis factor α, interleukin 1β and 6), in the PTZ-treated zebrafish larvae, were substantially suppressed by the alkaloid (Zhang et al., 2020).

Recently, our research group has demonstrated the anticonvulsant effect of the *Berberis sibrica radix* extract; berberine and another isoquinoline alkaloid (palmatine) were isolated from this extract and studied by means of zebrafish larvae PTZ-induced seizure assay (Gawel et al., 2020a). In our study, berberine was used as a reference compound. The *Berberis sibrica* root extract and both studied alkaloids decreased hyperlocomotion in the PTZ-treated larvae. The analysis of the LFP recordings from the larval optic tectum confirmed the anticonvulsant effect of the studied drugs. Both alkaloids reduced the number of PTZ-induced epileptiform-like discharges, but only berberine shortened the mean duration of epileptiform-like events. In contrast, palmatine reduced the *bdnf* and *c-fos* mRNA expression in the PTZ-exposed larvae (Gawel et al., 2020a).

In our study, a mixture of palmatine and berberine combined at their ED_16_ concentrations (palmatine and berberine at a 1:2.17 ratio that resembled their distribution in the plant extract), was also studied (Gawel et al., 2020a). A hyper-additive interaction between these two alkaloids was noted and the combination employed revealed an anticonvulsant effect that was comparable to the total *Berberis sibrica* root extract. Those results suggest that the mechanisms of the anticonvulsant action of palmatine and berberine are distinct and these two alkaloids are probably the main active compounds that determine the anticonvulsant properties of the *Berberis sibrica* extract (Gawel et al., 2020a).

### 2.2. Bioflavonoids

Naringenin [5,7-dihydroxy-2-(4-hydroxyphenyl)chroman-4-one] is a flavonoid formed due to the hydrolysis of its naringin or narirutin glycone forms. It is mainly present in grapefruit juice, but might also be found in other citrus fruit (Wilcox et al., 2006). Naringenin has many proven pharmacological effects, among others, antioxidant, anti-inflammatory, antiulcer, hypocholesterolemic, antimutagenic, and neuroprotective activity (Ribeiro, 2011).

Kaempferol [3,5,7-trihydroxy-2-(4-hydroxyphenyl)chromen-4-one], a flavonoid present in many vegetables and fruit, e.g., broccoli, cabbage, beans, tomato, strawberries, and grapes, can also be found in plants used in traditional medicine, e.g., in *Ginkgo biloba, Tilia* spp.*, Equisetum* spp., as well as in *Crinum jagus.* The last is commonly used in Cameroon as a traditional remedy for epilepsy, convulsions, and psychoses (Calderón-Montaño et al., 2011; Taiwe et al., 2016). Kaempferol has a wide range of pharmacological effects, *inter alia*, antioxidant, anti-inflammatory, neuroprotective, antidiabetic, antiosteoporotic, anxiolytic, analgesic and antiallergic activities (Calderón-Montaño et al., 2011).

In a study by Copmans et al. (2018) (Table 2), naringenin, kaempferol, and their methylated derivatives, i.e., naringenin 7-*O*-methyl ether, naringenin 4′,7-dimethyl ether and kaempferide (4’-*O*-methyl kaempferol), were investigated *via* zebrafish larvae PTZ-evoked hyperlocomotion assay. Here, naringenin 7-*O*-methyl ether and naringenin 4′,7-dimethyl ether were found to be the most efficient in attenuating seizure-like behavior, as they reduced PTZ-induced hyperlocomotion to *ca.* 20–30% of the control PTZ-evoked activity. Kaempferol, kaempferide, and naringenin had markedly weaker anticonvulsant effects. It cannot be excluded that differences in the activity of the studied drugs, especially in the case of naringenin, were due to their different bioavailability and insufficient time of incubation, i.e., 1 h was not long enough to penetrate the blood–brain barrier (Guarin et al., 2021).

To verify behavioral results, LFP recordings were obtained. Naringenin 7-*O*-methyl ether, naringenin 4′,7-dimethyl ether, and kaempferol were found to reduce the percentage of larvae with epileptiform-like activity in the LFP recordings. Additionally, the studied naringenin derivatives reduced the number and cumulative duration of the PTZ-induced epileptiform-like events. Since naringenin 7-*O*-methyl ether itself increased larval locomotion, in comparison to the vehicle-treated control group, the larvae pre-incubated in the studied compounds alone were also submitted to a LFP recordings assay. Interestingly, the researchers noted that naringenin 7-*O*-methyl ether, as well as naringenin alone, induced the epileptiform-like events recorded in the larval optic tectum. As a follow up, naringenin 4′,7-dimethyl ether was also investigated *via* intravenous (*iv*) PTZ and 6-Hz psychomotor seizure tests in mice. In both tests, this compound exhibited anticonvulsant properties. The results obtained in the zebrafish larvae and mouse tests suggest that naringenin 4′,7-dimethyl ether might be considered a new drug candidate for epilepsy treatment, although in-depth analysis to determine the mechanisms of its action is needed (Copmans et al., 2018).

### 2.3. Cannabis sativa

*Cannabis sativa* L. (Cannabaceae), also called “hemp” or “marihuana,” has been a commonly used medicinal plant since ancient times. It is believed that *Cannabis sativa* is native to the alpine foothills of the Himalayas. Nowadays, *Cannabis sativa* is cultivated all over the world and used in the food, textile, and pharmaceutical industries (ElSohly et al., 2017). Interest in using *Cannabis sativa* and cannabis-based products in epilepsy treatment has grown rapidly in recent years (Gaston and Friedman, 2017). One of the phytocannabinoids that are deprived of the psychoactive activity, i.e., CBD, was approved for the treatment of seizures in Dravet and Lennox–Gastaut syndrome patients (Morano et al., 2020).

Although the anticonvulsant properties of *Cannabis sativa* isolates have been known for centuries and used by local herbalists, and they have been confirmed in numerous studies using rodent models of seizures and epilepsy (Chesher and Jackson, 1974; Elisabetsky et al., 1999; Elisabetsky and Brum, 2003; Shirazi-zand et al., 2013; Gobira et al., 2015; Kaplan et al., 2017; Klein et al., 2017; Vilela et al., 2017; Patra et al., 2019; Anderson et al., 2020; Gray and Whalley, 2020; Zou et al., 2020; Costa et al., 2021; Supplementary Table 1), there are also some studies that evaluated the protective properties of selected phytocannabinoids using zebrafish larvae (Samarut et al., 2019; Thornton et al., 2020; Table 2).

Using zebrafish larvae to study the therapeutic properties of cannabis-derived medicaments is highly reasonable since all important endocannabinoid-related genes are expressed in the zebrafish larvae. Moreover, a high level of similarity between the cannabinoid system in zebrafish and other vertebrates makes zebrafish an appropriate model for studying the therapeutic properties and mechanism of action of phytocannabinoids (Rodriguez-Martin et al., 2007). The expression of cannabinoid receptor 1 (CB-1), the major target for the cannabinoids, in the zebrafish larvae begins as early as 24 hpf and its increased expression in the brain structures is observed by 48 hpf (Lam et al., 2006).

Samarut et al. (2019) employed two zebrafish larvae models of seizures, i.e., PTZ-induced seizure test and *gabra1^−/−^* zebrafish mutants (Table 1) to evaluate the single and combined effects of CBD and Δ-9-tetrahydrocannabinol (THC; Table 2). In the study, both CBD and THC alone substantially reduced the seizure-like activity in the PTZ-induced and genetic models. However, higher concentrations of the studied compounds had to be administered to reduce seizures in *gabra1^−/−^* mutants. The obtained results suggest that the anticonvulsant effect produced by the studied phytocannabinoids, especially by THC, might at least partially result from their sedative activity. The co-exposure of both drugs tested at their low ineffective concentrations, at a 1:1 ratio, resulted in a synergistic anticonvulsant effect in both models (Samarut et al., 2019).

In a study by Thornton et al. (2020) (Table 2), five cannabis-derived compounds, i.e., CBD, THC, cannabidivarin, cannabinol, and linalool, were investigated using PTZ-induced seizure model and *scn1Lab^−/−^* zebrafish mutants (Table 1). This study confirmed the results that had been previously presented by Samarut et al. (2019) on the anticonvulsant effect of CBD and THC in the PTZ-induced seizure test. However, there were some differences concerning the effective concentrations of the studied compounds, especially CBD, which was effective at relatively low concentrations in comparison to the results obtained by Samarut et al. (2019). These differences might have resulted from differences in time exposure to the studied compounds—in the study presented by Samarut et al. (2019), zebrafish were incubated for 1 h, while in the study conducted by Thornton et al. (2020), the incubation time was 24 h. Cannabidivarin, cannabinol, and linalool did not affect seizure-like activity in the zebrafish larvae PTZ-induced seizure assay (Thornton et al., 2020). Moreover, CBD and THC reduced the seizure activity in the *scn1Lab^−/−^* zebrafish larvae. The anticonvulsant effect of cannabinol and linalool was also noted in the *scn1Lab^−/−^* mutants. While the efficacy of CBD in Dravet syndrome models had been known earlier, the efficacy of cannabinol and linalool was demonstrated in this study for the first time (Thornton et al., 2020).

### 2.4. Curcuma longa

*Curcuma longa* L. (Zingiberaceae; turmeric) is a herb native to South Asia. Due to its intensive flavor and yellow color, turmeric rhizome became an important spice in the kitchens of South Asia, Iran, China, Polynesia, and Thailand. For at least 2,500 years, turmeric has also been a common and widely used herbal constituent in traditional South Asia and Chinese medicine as a remedy for plenty of diseases. The main active constituent of turmeric is curcumin (2–5%) which possesses antioxidant, antimicrobial, anti-inflammatory, antiangiogenic, antimutagenic, and antiplatelet properties (Kocaadam and Şanlier, 2017). Recent studies have also demonstrated the anticonvulsant properties of curcumin (Agarwal et al., 2011; Reeta et al., 2011; Ahmad, 2013; Akula and Kulkarni, 2014; Arbabi Jahan et al., 2018; Dhir, 2018; Supplementary Table 1), though the clinical relevance of this indication is doubtful due to very low absorption of curcumin from the gastrointestinal tract (Lopresti, 2018). However, it is very likely that curcumin is not the only constituent of *Curcuma longa* with anticonvulsant properties because another relevant constituent of turmeric—turmeric oil, shows some therapeutic properties which also include neuroprotective and antiseizure activities (Dohare et al., 2008; Zhang L. et al., 2017; Supplementary Table 1).

Orellana-Paucar et al. (2012) (Table 2) revealed the anticonvulsant activity of the methanolic extract of turmeric utilizing the larval zebrafish PTZ-induced seizure model. To identify active constituents of turmeric, a curcuminoids mixture (98% of curcumin) of turmeric and turmeric oil was also examined. They found that the curcuminoids mixture was more efficient in attenuating PTZ-induced seizure behavior than turmeric oil alone, which revealed an anticonvulsant effect only at the highest concentration tested. Interestingly, both the curcuminoids mixture and turmeric oil alone slightly increased zebrafish locomotor activity compared to the control group (Orellana-Paucar et al., 2012).

Active constituents of turmeric oil, i.e., *ar*-turmerone*, α,β*-turmerone, and *α*-atlantone, also displayed anticonvulsant action, but they increased the locomotor activity in the control zebrafish. To verify whether turmeric oil itself has proconvulsant activity, LFP recordings were obtained. The analysis revealed that it did not cause any epileptiform-like discharges and it reduced the number and duration of ictal-like events in the PTZ-exposed larvae. Finally, stage of the study, the anticonvulsant potential of turmeric oil and its two main constituents, i.e., *ar*-turmerone and *α,β*-turmerone, was confirmed *via* timed *iv* PTZ test in mice (Orellana-Paucar et al., 2012).

To sum up, the study conducted by Orellana-Paucar et al. (2012) revealed, for the first time, that the anticonvulsant properties of *Curcuma longa* are not only due to curcumin presence, but they also depend on other compounds, i.e., *ar*-turmerone, *α,β*-turmerone, and *α*-atlantone. Importantly, in this study, the effects of the turmeric-derived compounds tested were verified carefully using both behavioral studies in zebrafish larvae, as well as LFPs analysis, and they involved a conventional seizure model in mice.

Since low bioavailability limits the therapeutic utility of curcumin (Lopresti, 2018), Bertoncello et al. (2018) (Table 2) used the micronized form of this compound that was prepared by supercritical carbon dioxide processing. The effect of the micronized curcumin in the PTZ-induced seizure tests in larvae and adult zebrafish was compared to the effect of curcumin in its natural form, as well as to valproic acid (classic ASM). In adult zebrafish, the studied compounds (administered intraperitoneally) exhibited some protective activity, but only valproate and micronized curcumin reduced the occurrence of tonic–clonic-like seizures. This paper demonstrates some interesting data, but the final confirmation of the superior anticonvulsant activity of micronized curcumin over curcumin must be checked in an LFP assay since the seizure score assay in larval zebrafish, as employed in this paper, is of low objectivity. It would also be recommended to investigate whether, and to what extent, the micronized form of curcumin is able to penetrate biological membranes better than curcumin.

Choo et al. (2021) (Table 2) analyzed the anticonvulsant properties of new synthetic analogs of curcumin using PTZ-induced hyperlocomotion assay as an initial screening platform. Among the 68 analogs tested, 15 substantially reduced the total distance traveled by and/or the maximum acceleration of PTZ-exposed larvae. The effective compounds were then tested in two zebrafish larvae genetic models of epilepsy, i.e., *gabra1^−/−^* and *gabrg2^−/−^* (Table 1). In the study, three of the selected derivatives of this polyphenol abrogated the behavioral manifestation of seizures in *gabra1a^−/−^* mutants, six of them showed anticonvulsant effect in *gabrg2^−/−^* zebrafish, and only one was effective in both genetic models. To complement behavioral observations in mutants, hit compounds were additionally tested in a transgenic reporter line, i.e., *Tg[neurod:GCaMP6f]*, which allowed detecting neuronal firing. Here, mutants and wild-type larvae were first incubated in curcumin derivatives and then laser stimuli were used to evoke the excitation of post-mitotic neurons in the larval optic tectum. Unfortunately, none of the selected analogs was able to rescue the neuronal firing in *gabra1a^−/−^* mutants and only two of them decreased fluorescence intensity upon laser stimuli in *gabrg2^−/−^* larvae (Choo et al., 2021).

In conclusion, the obtained data have confirmed that the PTZ-induced seizure assay is a good standard for the initial screening of compounds, but it may yield false-positive results. Thus, e.g., neuronal firing assay or LFP recordings, should be performed. Nonetheless, the paper by Choo et al. (2021) revealed the anticonvulsant potential of compounds that were synthesized based on the chemical structure of naturally occurring compounds.

### 2.5. Garcinia oligantha

*Garcinia oligantha* Merr. (Guttiferae) is a shrub commonly present in the forests of the Guangdong and Hainan provinces in China. In traditional folk medicine, *Garcinia oligantha* is used for body detoxification and as a remedy for inflammation (Lin F. et al., 2021). Moreover, cytotoxic properties of *Garcinia oligantha* extracts and xanthones derived from this plant have been reported (Tang et al., 2016). Interestingly, there are no reports regarding the use of this plant in traditional medicine for epilepsy treatment or experimental studies reporting its anticonvulsant properties.

Gong et al. (2020) (Table 2) studied the anticonvulsant properties of three xanthones isolated from *Garcinia oligantha* leaves, i.e., oliganthin H, oliganthin I, and oliganthin N. The compounds were evaluated *via* PTZ-induced seizure test in zebrafish larvae. Of the three compounds tested, the strongest anticonvulsant effect was produced by oliganthin H—the lower concentrations tested prolonged seizure latency, while the highest concentration tested additionally reduced the total distance traveled. A substantial reduction of PTZ-induced seizure-like activity was also caused by oliganthin N, though only at the maximum concentration tested. Oliganthin I did not inhibit PTZ-induced activity, but was able to prolong seizure latency. Since oliganthin H was the most potent compound, its anticonvulsant activity was precisely analyzed. Quantitative reverse transcription PCR (RT-qPCR) analysis revealed that it normalized the expression of some genes related to the neuronal activity, GABA, and glutamate neurotransmission, i.e., *npas4a*, *c-fos*, *pyya*, *bdnf, gabra1, gad1, glsa*, and *glula* genes. Moreover, a molecular docking study demonstrated that oliganthin H has a strong binding potency toward the GABA_A_ receptor and this complex is characterized by good stability. The obtained results indicate that the anticonvulsant effect of oliganthin H involves the GABA/glutamate system (Gong et al., 2020).

### 2.6. Indigofera arrecta

*Indigofera arrecta* Hochst. ex A.Rich. (Natal indigo) is a member of the *Leguminaceae* family and it naturally occurs in savannah regions. It is found, *inter alia*, in Tropical Africa, Saudi Arabia, Eastern and Southern Africa. The great availability of this plant in Africa results in its intensive use by traditional healers, e.g., as a soothing agent for venomous insect and snake bites, and as an antiseptic, healing, and antipruritic agent (Gerometta et al., 2020). There are also reports regarding the use of phytomedicines derived from *Indigofera arrecta* in the treatment of epilepsy (among other places, in the Congo), anxiety and other nervous system diseases (Amponsah, 2014; Aourz et al., 2019; Table 2). Indirubin [3-(3-oxo-1H-indol-2-ylidene)-1H-indol-2-one], a compound naturally present in *Indigofera arrecta*, acts as an inhibitor of glycogen synthase kinase-3 (GSK-3); (Aourz et al., 2019), cyclin dependent kinases type 1, 2, and 5 (Canavese et al., 2012), as well as an activator of the aryl hydrocarbon receptor (AhR); (Adachi et al., 2001).

Aourz et al. (2019) (Table 2) demonstrated the anticonvulsant activity of the methanolic extract obtained from *Indigofera arrecta* leaves in the larval zebrafish PTZ-induced seizure assay. Subsequently, indirubin, an active metabolite, was isolated and evaluated in the seizure test. It was found to reduce PTZ-induced hyperlocomotion, as well as the duration of epileptiform-like activity and the number of interictal and ictal-like spikes (Aourz et al., 2019). Moreover, rodent models of seizures and epilepsy, namely the 6-Hz-induced psychomotor seizure and *iv* PTZ tests in mice, as well as the pilocarpine model of epilepsy in rats, were applied to evaluate the anticonvulsant effect of indirubin (Supplementary Table 1). Although it did not show anticonvulsant activity in the *iv* PTZ test in mice, it dose-dependently prevented 6-Hz- and pilocarpine-induced seizures. The experiments in which a panel of compounds with different known affinities toward GSK-3, cyclin dependent kinases type 1, 2, and 5, as well as AhR, was used pointed out GSK-3 as a target in the mechanisms of anticonvulsant action of indirubin. To further strengthen these findings, Aourz et al. (2019) induced transient knockdown of *gsk-3ß* in larval zebrafish using an antisense morpholino oligomer, and showed that the lack of *gsk-3ß* exhibited a protective effect against PTZ-evoked seizures.

A study conducted by Aourz et al. (2019) is particularly relevant for a few reasons. First, zebrafish larvae were used as an animal model to point out, for the first time, GSK-3 as a new target for epilepsy treatment. Using a morpholino oligomer for the transient knockdown of *gsk-3ß* in larval zebrafish, the authors proved the hypothesis concerning the target for indirubin. Different inter-species models of seizures were combined, and the utility of larval zebrafish for the initial screening of plant metabolites with potential anticonvulsant activity was highlighted.

### 2.7. Magnolia officinalis

Bark extracts of different magnolia species, including *Magnolia officinalis* Rehder & Wilson (Magnoliaceae), have been used in traditional Chinese and Japanese medicine. Health-promoting properties of *Magnolia officinalis* include mainly antioxidant and anti-inflammatory action, but sedative, anxiolytic, antidepressant and antiepileptic activities have also been reported (Martínez et al., 2006; Chen et al., 2011; Lee et al., 2011; Han et al., 2015; Poivre and Duez, 2017; Vega-García et al., 2019). The two major biologically active compounds of the *Magnolia officinalis* bark are magnolol and honokiol—structurally related neolignans with similar pharmacokinetics. Both compounds display antiseizure properties, and exert GABAergic and cannabimimetic activity (Alexeev et al., 2012; Chen et al., 2012; Woodbury et al., 2013; Sarrica et al., 2018; Lin Y. et al., 2021; Supplementary Table 1).

Anticonvulsant properties of the *Magnolia officinalis* bark extracts and their active compounds, i.e., magnolol and honokiol, were tested both in the larval zebrafish PTZ-induced hyperlocomotion assay (Moradi-Afrapoli et al., 2017; Li et al., 2020; Table 2) and in the ethyl ketopentenoate (EKP) model of pharmacoresistant seizures (Zhang Y. et al., 2017; Table 1). A study by Moradi-Afrapoli et al. (2017) revealed that the ethyl acetate extract of the *Magnolia officinalis* bark concentration-dependently reduced the PTZ-induced seizure-like behavior. Magnolol was identified as the main ingredient of the extract, and a significant anticonvulsant effect in the PTZ-induced seizure assay was noted for this compound (Moradi-Afrapoli et al., 2017).

Li et al. (2020) studied three extracts (i.e., water, ethanol, and acetone) from the *Magnolia officinalis* bark in the PTZ-induced seizure test in the zebrafish larvae. Only two, i.e., ethanol and acetone extracts, revealed concentration-dependent anticonvulsant effects. Those two extracts also effectively reduced EKP-induced seizure-like behavior. The active ingredients of *Magnolia officinalis*, i.e., magnolol and honokiol, brought about a substantial (over 40%) reduction in PTZ-induced seizure movement at the highest concentrations tested, while in the EKP-induced seizure test, both compounds displayed concentration-dependent anticonvulsant effects at all concentrations tested. In addition to the behavioral results, both magnolol and honokiol reduced the frequency, as well as duration of epileptiform-like discharges in the PTZ- or EKP-treated zebrafish larvae. Power spectral density analysis of electrophysiological signals also revealed that these compounds attenuated PTZ- and EKP-increased LFP power. A study presented by Li et al. (2020) not only confirmed previous reports concerning the anticonvulsant effects of *Magnolia officinalis* extracts and their ingredients in the zebrafish larvae PTZ-induced seizure assay (Moradi-Afrapoli et al., 2017), but also complimented them by the data from the EKP-induced seizure test, which is thought to be an acute model of pharmacoresistant seizures (Zhang Y. et al., 2017). Of note, the results obtained in the behavioral tests were confirmed by an in-depth analysis of electrophysiological signals (Li et al., 2020).

### 2.8. Pterostilbene

Pterostilbene [4-(3,5-dimethoxystyryl)phenol] is a stilbenoid—a naturally dimethylated analog of resveratrol. Originally, it was isolated from sandalwood and was later also identified in blueberries and grapes. Pterostilbene has numerous health-promoting properties, including anti-inflammatory, antioxidant, antitumor, neuroprotective, and antidiabetic activity (Chang et al., 2012; Teng et al., 2021). Recent studies have suggested its influence on cognition, anxiety-like behavior, and other neuronal functions (Al Rahim et al., 2013; Poulose et al., 2015).

Our research team has demonstrated the anticonvulsant activity of pterostilbene in the larval zebrafish PTZ-induced seizure assay (Nieoczym et al., 2019; Table 2). Pterostilbene substantially limited PTZ-induced hyperlocomotion and reduced both the total and mean duration of the epileptiform-like events registered from optic tectum (Nieoczym et al., 2019). The anticonvulsant effect of pterostilbene was confirmed in three acute seizure tests in mice, i.e., in the maximal electroshock seizure (MES) threshold test, the psychomotor 6 Hz-induced seizure threshold test, as well as the *iv* PTZ test (Supplementary Table 1). The mechanism of anticonvulsant action of ptereostilbene has not been explained yet (Nieoczym et al., 2019).

### 2.9. Solanum torvum

*Solanum torvum* Tw. (Solanaceae) is a shrub commonly found in South India, Malaysia, China, Philippines, Thailand, West Indies, and Tropical America (Balachandran et al., 2015). In Thailand, it is known as “Turkey berry” and in India as “wild brinjal” (Yuan et al., 2016; Vanti et al., 2020). In India and Africa, *Solanum torvum* is cultivated for its edible fruit. The fruit, leaves, and roots of this plant are commonly used in traditional Cameroonian and Chinese medicine as a remedy for fever, hypertension, gastralgia, or furuncle (Mohan et al., 2009; Yuan et al., 2016). Antiviral, antioxidant, analgesic and anti-inflammatory activities of extracts prepared from this plant have been demonstrated (Balachandran et al., 2015).

Three different formulations, i.e., the aqueous extract, the methanol crude extracts and traditional water decoction from the aerial parts of *Solanum torvum* revealed anticonvulsant activity in the PTZ-induced seizure assay in larval zebrafish (Challal et al., 2014; Table 2). Since the composition of methanolic and aqueous extracts is very similar to the preparations used in traditional herbal medicine, findings from the seizure test justify the use of *Solanum torvum* extracts in epilepsy treatment.

In the composition of the methanolic extract, some steroid glycosides were found and six ingredients were identified, i.e., tervoside J, L and K, paniculonin B and A, and (22*R*,23*S*,25*S*)-3*β*,6*α*,23-trihydroxy-5*α*-spirostane-6-*O-β*-dxylopyranosyl-(1 → 3)-*O-β-D*-quinovopyranoside. These compounds, as well as solanolide (an aglycone obtained by the acid hydrolysis of the methanolic extract), were also evaluated for anticonvulsant activity. Substantial and concentrations-dependent anticonvulsant action was reported for torvoside J, while torvoside L and K only induced a tenuous effect (Challal et al., 2014).

An important advantage of the study conducted by Challal et al. (2014) is the detailed analysis of the chemical composition of *Solanum torvum* extracts and the identification of biologically active compounds. However, the anticonvulsant activity of the studied extracts and compounds was identified only through evaluating zebrafish larvae behavior and was not verified by any deeper phenotyping.

### 2.10. *Zingiber purpureum* and *Zingiber officinale*

Different species of the *Zingiberacae* family have been used as herbal medicines since ancient times. Plants belonging to the genus *Zingiber* (ginger) are present mainly in India, Japan, China, South Korea, Indo-China, and Southeast Asia, and they are especially popular in these regions. Phenolic acids and terpene compounds are the main active ingredients of *Zingiber* spp. and are responsible for their pharmacological and biological properties. Ginger extracts have revealed antimicrobial, antioxidant, anti-inflammatory properties, they are also used to attenuate motion sickness, gastrointestinal and menstrual disorders, asthma and headache (Gupta and Sharma, 2014; Kukula-Koch et al., 2018; Khan et al., 2019; Shahrajabian et al., 2019; Kausar et al., 2021).

Extracts from two plants from the genus *Zingiber*, i.e., *Zingiber purpureum* Ridl. and *Zingiber officinale*, Rosc. were studied in the zebrafish larvae PTZ-induced seizure test (Brillatz et al., 2020; Gawel et al., 2021; Table 2). Of the four *Zingiber purpureum* extracts tested, i.e., hexane, ethyl acetate, ethanol, and aqueous, the hexane extract exerted the most potent anticonvulsant effect in PTZ-treated zebrafish larvae. Fifteen fractions obtained from this extract were evaluated in the zebrafish larvae PTZ assay and one, a mixture of *trans*-banglene and *cis-*banglene, showed an especially potent anticonvulsant effect, as it reduced PTZ-induced hyperlocomotion by as much as 68%. When tested separately, the strongest anticonvulsant effect was noted for *cis-*banglene. Interestingly, this compound revealed a 4–8 times more potent anticonvulsant effect than topiramate (a broad-spectrum ASM). The mixture of *cis-*bangelene and *trans-*banglene (50/50 ratio) decreased both the seizure score and mortality in the PTZ-induced seizure test in mice (Brillatz et al., 2020).

Studies of the anticonvulsant potential of plants belonging to the genus *Zingiber* have been recently expanded by our research group (Gawel et al., 2021). We noted that methanolic extract from the *Zingiber officinale* rhizome reduced PTZ-induced hyperlocomotion in the zebrafish larvae. Subsequently, 6-gingerol was identified as the active ingredient of the studied extract and was separately evaluated. We found that it reduced the seizure-like behavior and decreased the number and mean duration of epileptiform-like events in the LFP recordings. Biochemical and molecular studies that were conducted to find the possible mechanism of anticonvulsant action of 6-gingerol also revealed that it affected mainly glutamatergic neurotransmission. Primarily, it lowered the glutamate level in the PTZ-treated zebrafish larvae and thus restored the glutamate/GABA ratio and balance between the excitatory and inhibitory neurotransmission in the brain. Secondly, 6-gingerol decreased the expression of the *grin2b* gene that encodes the subunit of the N-methyl-D-aspartate (NMDA) receptor and thus additionally mitigated excitatory glutamatergic neurotransmission. Molecular docking analysis showed that the studied compound might also interact with the ATD- and glutamate-binding site, as well as within the ion channel in the NMDA receptor complex (Gawel et al., 2021).

The studies presented by Brillatz et al. (2020) and by our group (Gawel et al., 2021) provide evidence for the anticonvulsant properties of ginger extracts and their active ingredients. We also aimed to verify the possible mechanisms of 6-gingerol anticonvulsant activity.

## 3. Studies regarding anticonvulsant effects of plant-derived drugs in zebrafish *vs.* rodents—a comparison

In Table 3, we have selected and gathered data from Table 2 (zebrafish assays) and Supplementary Table 1 (rodent assays) to compare zebrafish *vs.* rodent studies. Most of the experiments on the anticonvulsant effect of plant metabolites were carried out in the PTZ model, both in zebrafish and rodents. Therefore, in this review, the zebrafish *vs.* rodent comparison refers primarily to PTZ-induced seizures. Generally, the results obtained from studies utilizing zebrafish and mouse models are qualitatively comparable at least in most of the cited reports. Consistent anticonvulsant effects were found for *ar*-turmerone, *α,β*-turmerone, CBD, curcumin, kaempferol, magnolol, naringenin 4′,7-dimethyl ether and pterostilbene (for details, see Table 3). However, it is worth emphasizing that only the CBD and curcumin results are based on studies conducted in different independent laboratories. In many others, these are often only single investigations, the results of which have not been validated by other authors, which makes it challenging, at least at this state of knowledge, to draw unequivocal conclusions and to make categorical recommendations. Incompatibility is noted for berberine, which is active in the PTZ-induced seizure model in zebrafish and ineffective in rodent PTZ assays. Inconsistencies were also noted for indirubin, linalool and naringenin, with only single reports available, which again makes it difficult to draw generalized conclusions. The reason for these differences is unknown and further research is needed.

**Table 3:** Anticonvulsant effects of plant-derived drugs in different seizure or epilepsy models in zebrafish and rodents—a comparison.

Drug	Species	Chemically induced convulsions/seizures/epilepsy tests/models								Electrically induced seizure tests		Genetic models
		PTZ	EKP	KA	Li-PILO/PILO	4-AP	NMDA	Quinolinic acid	Cocaine	6 Hz	MES	
*ar*-turmerone	Danio	+ (BEH)										
	Mice	+ (BEH)								+ (BEH)		
		+ (BEH)										
*α,β*-turmerone	Danio	+ (BEH)										
	Mice	+ (BEH)										
Berberine	Danio	+ (BEH)										
		+ (BEH and LFP)										
		+ (BEH and LFP)										
	Mice/rats	*NS* (BEH)		+ (BEH)	+ (BEH)	+ (BEH)					+ (BEH)	
		*NS* (BEH)		+ (BEH)							*NS* (BEH)	
		*NS*/+ (BEH)										
CBD	Danio	+ (BEH)										+ (BEH)
		+ (BEH)										+ (BEH)
	Mice/rats	+ (BEH)			+ (BEH)				+ (BEH)	+ (BEH)	+ (BEH)	+ (BEH)
		+ (BEH)									+ (BEH)	
		+ (BEH)								+ (BEH)	+ (BEH)	
		+ (BEH)							+ (BEH)		+ (BEH)	
		+ (BEH)								*NS* (BEH)	+ (BEH)	
		+ (BEH)									+ (BEH)	
											+ (BEH)	
Curcumin	Danio	+ (BEH)										+ (BEH)
		+ (BEH)										NS (neuronal activity)
	Mice/rat	+ (BEH)			+ (BEH)						+ (BEH)	
		+/*NS* (BEH)										
		+ (BEH)										
		+ (BEH)										
Honokiol	Danio	+ (BEH and LFP)	+ (BEH and LFP)									
	Mice						+ (BEH)					
							+ (BEH)					
Indirubin	Danio	+ (BEH and LFP)										
	Mice/rat	NS (BEH)			+ (BEH)					+ (BEH)		
Kaempferol	Danio	+ (BEH and LFP)										
	Mice	+ (BEH)										
LN	Danio	*NS* (BEH)										+ (BEH)
	Mice	+ (BEH)					+/*NS* (BEH)	+ (BEH)			*NS* (BEH)	
		*NS* (BEH)										
Magnolol	Danio	+ (BEH and LFP)	+ (BEH and LFP)									
	Mice	+ (BEH and EEG)					+ (BEH)					
Naringenin	Danio	– (BEH and LFP)										
	Mice	+ (BEH)		+ (BEH)	*NS* (BEH acute)						+ (BEH)	
		+ (BEH)			+ (BEH chronic)							
Naringenin 4′,7-dimethyl eter	Danio	+ (BEH and LFP)										
	Mice	+ (BEH)								+ (BEH)		
Pterostilbene	Danio	+ (BEH and LFP)										
	Mice	+ (BEH)								+ (BEH)	+ (BEH)	
THC	Danio	+ (BEH)										+ (BEH)
		+ (BEH)										+ (BEH)
	Mice	*NS* (BEH)							+ (BEH)			+ (BEH)
												NS (BEH)

4-AP, 4-aminopyridine; BEH, behavior; CBD, canabidiol; EEG, electroencephalography; EKP, ethylketopentenoate; KA, kainic acid; Li-PILO, lithium-pilocarpine; LFP, local field potential; LN, linalool; MES, maximal electroshock seizure; NMDA, N-methyl-D-aspartate; NS, non-significant; PILO, pilocarpine; PTZ, pentylenetetrazole; THC, delta(9)-tetrahydrocannabinol. A+ and − sign indicates an anticonvulsant (positive) and proconvulsant (negative) effect reported in a single publication. Since the results of studies are not identical this labeling system makes it possible to assess whether an antiepileptic effect has been reported in the majority of studies. Data extracted from Table 2 and Supplementary Table 1.

It is interesting (and surprising) to compare the effectiveness of plant metabolites in the zebrafish PTZ-induced seizure model *vs.* electrically induced seizures (6 Hz and MES) in rodents because there is an almost perfect agreement of all analyzed plant substances. The only exception is naringenin, though it is the only single result (Table 3). It is worth noting that such a high compatibility applies to both anticonvulsant activity and the lack of anticonvulsant effect. If it is confirmed in a larger group of plant metabolites, it could be predicted that the PTZ-induced seizure model in zebrafish might replace the PTZ test and the electric convulsion tests performed on rodents as a screening platform.

A meaningful advantage of zebrafish lies in the ease, compared to rodents, of obtaining genetically altered strains. A comparison of the anticonvulsant effect of the analyzed plant metabolites in genetic models *vs.* PTZ and electric convulsions indicates a very high convergence of the results obtained in zebrafish and rodents, which additionally provides a positive validation of the genetic models in zebrafish for high-throughput screening purposes.

## 4. Methodology: challenges

For the past 15 years, a tremendous increase in the use of zebrafish in biomedical sciences has been observed. Epilepsy was one of the first central nervous system diseases that were studied using zebrafish as a model. Baraban et al. (2005) were first to describe the PTZ-induced seizure assay in zebrafish larvae, and Orellana-Paucar et al. (2012) were one of the first to employ this test for the screening of plant constituents. Since then, a number of new plant-derived anticonvulsant drug leads have been discovered (Bertoncello and Bonan, 2021). The PTZ-induced model of seizures described by Baraban et al. (2005) is still widely used as a first-choice screening platform. However, a discovery of Yaksi et al. (2021) shed light on problems in its application. In this paper, the authors pointed out that relying only on the locomotor assessment as a readout of anticonvulsant activity may, in a small number of cases, yield false-positive results. We, therefore, recommend evaluating hit molecules from the group of screened compounds by means of different complementing techniques—ideally, LFPs recorded from the larval optic tectum (Baraban et al., 2005; Afrikanova et al., 2013; Gawel et al., 2020b,c) or assessing neuronal activity using bioluminescence, for example, employing transgenic zebrafish expressing fluorescent photoprotein GFP-apoAequorin [*Tg(elavl3:eGFP-apoAequorin)*; Zhang Y. et al., 2017]. Alternatively, transgenic lines encoding calcium indicators [e.g., Tg(elavl3:GCaMP6s)] may be used for this purpose (Choo et al., 2021).

Confocal imaging of GCaMP expressing zebrafish larvae with LFP recording has been applied to study epileptic-like events in the whole brain (Kettunen, 2020). Monitoring the neuronal network for dynamic activities provides an important understanding of seizure initiation and propagation. A recent study has revealed the rapid propagation of seizure activity from anterior-to-posterior brain regions using GCaMP imaging in the central nervous system of zebrafish, which was performed in zebrafish brains treated with PTZ, and which was also observed for its behavior activity and LFP recording (Liu and Baraban, 2019).

Calcium imaging has also become a very useful tool, similar to brain LFP recordings, to assess the level of neuronal firing in epileptic and normal zebrafish brains. It was used to record neuronal activity in *gabrga1* and *gabrg2* mutant zebrafish brains (Samarut et al., 2018; Liao et al., 2019). However, if none of the above-mentioned methods is feasible, at least the *c-fo*s oncogene (a marker of neuronal activity) or epilepsy-specific marker (e.g., *npas4* or *sestrin*) expression in zebrafish brain using RT-qPCR (Klarić et al., 2014; Johnson et al., 2015; Shan et al., 2018; Zhang et al., 2020; Gawel et al., 2020a) or whole-mount *in situ* hybridization (Zheng et al., 2018) should be performed. For adult zebrafish, RT-qPCR (Mazumder et al., 2019; Gawel et al., 2020c) or whole-mount *in situ* hybridization (Ariyasiri et al., 2021) of such genes can be undertaken.

One should remember that the PTZ-induced seizure assay in zebrafish is considered to be an equivalent of tonic–clonic-like seizures in humans. Thus, other pharmacological models should be applied for initial screening purposes. As mentioned, some have been occasionally used (e.g., pilocarpine or picrotoxin) in larval zebrafish (Gawel et al., 2020b), but they have not been employed for high-throughput screening purposes so far. Therefore, the validation of these chemical models is mandatory. At the same time, the EKP-induced seizure model, which is considered to be a model of pharmacoresistant epilepsy (Zhang Y. et al., 2017), has been recently successfully applied by Li et al. (2020) and pointed out magnolol and honokiol as compounds with anticonvulsant potency. Nevertheless, one should bear in mind that EKP is not commercially available. Despite its limited use so far, it has the potential to become a standard chemoconvulsant for the initial screening of extracts/compounds, comparable to the PTZ assay.

Another challenge that must be met is the need to implement zebrafish genetic models of epilepsy as routine, commonly available models to expand screening possibilities. Until now, many mutations in genes have been identified as causing epilepsy, but the genotype–phenotype correlation has not been well understood (Hortopan et al., 2010). Some research groups have developed zebrafish lines carrying mutations in epilepsy-causing genes, showing that mutant zebrafish undergo specific types of spontaneous seizures under stress conditions (Copmans et al., 2017). The GABA receptor loss of function α and γ subunit produce light reflex seizures in zebrafish larvae. As researchers were studying epilepsy behavior in mutant zebrafish, they surprisingly learned that the different mutations in the same gene produced different types of seizures under light (stress) condition (Samarut et al., 2018). This shows that not all mutations produce the same type of epilepsy, and each type of epilepsy requires an appropriate personalized ASMs treatment (Liao et al., 2019). Precision medicine is a therapeutic approach that is individually selected for each patient and that ideally targets molecular pathomechanisms of the disease (Balestrini et al., 2021). In our understanding, epilepsy offers an excellent, yet extraordinarily challenging, opportunity for treatment personalization, given the very large pool of genes which have been discovered in the past decade (Nabbout and Kuchenbuch, 2020). This modification, which is now possible in zebrafish, proves to be an easy high-throughput drug screen which can now be possible for each genetic phenotype (Cunliffe et al., 2015). For example, Thornton et al. (2020) used a zebrafish model of Dravet syndrome (*scn1Lab^−/−^*) whereas Choo et al. (2021) employed *gabra1a* and *gabrg2* knockouts to point out some cannabis constituents or curcumin analogs with antiseizure activity, respectively. All the above-mentioned mutants are in fact very good for initial screening purposes because of their clear phenotype: all those mutants have disturbed basic or stimulated (light) locomotor activity, and compounds with anticonvulsant activity counteract the behavioral impairments.

Another important issue which should be considered is the use of appropriate solvents or carriers for plant extracts or isolated molecules. This requires special attention because most molecules do not dissolve in water. If the molecule of interest is not soluble in the water, in most cases scientists, use dimethyl sulfoxide (DMSO) as a polar solvent. It has been shown that up to 2–2.5% DMSO was safe for embryos or larval zebrafish of the AB strain (Maes et al., 2012), albeit its safety was based only on morphological descriptors and one zebrafish line. A concentration of 1% DMSO is usually used for screening (Copmans et al., 2018; Aourz et al., 2019; Brillatz et al., 2020), but for genetic lines, it may be decreased to 0.1%. As a rule of thumb, the lowest possible concentration should always be used. This is especially true taking into account the data showing that DMSO itself induces seizures in both rodents (Kovács et al., 2011) and humans (Bauwens et al., 2005; Maral et al., 2018). It cannot be excluded that higher concentrations of DMSO in larval zebrafish might affect LFP recordings, thus, it is highly recommended to use as low concentrations as possible to avoid this interference.

Alternatively, if the molecule of interest is not soluble in DMSO, other solvents, e.g., ethanol, might be used with caution. It was found, however, that ethanol in intermediate and high concentrations produced hyper- and hypoactivity of larvae (Lockwood et al., 2004; Guo et al., 2015), respectively, which can interfere with the final outcome derived from the locomotor activity assessment of PTZ-induced seizures—leading to false-positive results and incorrect conclusions. Another solvent, acetone, is also contraindicated since it has been found to exert anticonvulsant activity in rodents (Likhodii et al., 2003). However, this has not yet been analyzed in zebrafish. Acetonitrile has been shown to be very toxic for larvae, while polyethylene glycol (PEG-400), methanol and propylene glycol have been found to be quite well tolerated (Maes et al., 2012). Furthermore, methanol did not affect the locomotor activity of larvae up to 1.5% (Lockwood et al., 2004). Nevertheless, all the above-mentioned solvents, except for DMSO, have been used occasionally, so they should be approached with caution.

At this moment, there is less data regarding drug pharmacokinetics in zebrafish than in rodents. There are, however, some scarce pharmacokinetic data for the known ASMs in adult zebrafish (Pieróg et al., 2021a,b). Nevertheless, most screening experiments, for obvious practical and ethical reasons, have been conducted in larval zebrafish. In those studies, zebrafish larvae have been usually exposed to the tested compounds for 18 h (Challal et al., 2014; Aourz et al., 2019; Brillatz et al., 2020) or for longer periods (Gawel et al., 2020a, 2021; Thornton et al., 2020), or sometimes for 2–3 h (Moradi-Afrapoli et al., 2017; Nieoczym et al., 2019; Choo et al., 2021). Guarin et al. (2021) compared the pharmacokinetics of different fluorescent dyes in zebrafish embryos after immersion or intra-yolk microinjection. Here, they found out that the intrabody absorption of dyes depended on the lipophilicity of the molecule, i.e., the more lipophilic compound, the better absorption. For less lipophilic molecules, microinjections seem to give better results. Since the lipophilicity of the investigated compound may strongly affect the outcome due to low brain penetration, in the case of less lipophilic compounds, longer incubation time is required (at least 24 h or preferably 48 h). Therefore, it seems reasonable to prolong the incubation time in larval zebrafish.

Thus far, most of the published data have focused on searching for new drug leads of plant origin without any follow up. However, first attempts have appeared to give insight into the molecular mechanisms of action of those “hits.” In larval zebrafish, the most frequently used technique for the initial evaluation of the mechanisms of action is RT-qPCR (Orellana-Paucar et al., 2013; Gong et al., 2020; Zhang et al., 2020; Gawel et al., 2021). Among the investigated genes, protooncogene *c-fos,* a general marker of neuronal activity, is one of the most often studied. However, there are some discrepancies between laboratories in terms of the duration of PTZ exposure before its overexpression is observed. Some authors have observed changes after 30 min (Gong et al., 2020; Zhang et al., 2020), others after 45 min (Gawel et al., 2020a) or 90 min (Gawel et al., 2021) of PTZ exposure. Interestingly, changes observed after 3 min (Zhang et al., 2020) were also described. One should consider that c-*fos* is expressed earlier than other markers, thus longer incubation in PTZ seems to be better for other genes. It is known that protein analysis is superior to the analysis of gene expression, but some antibodies available in the market are not dedicated to zebrafish, which might be one of the reasons why RT-qPCR continues to be more often conducted (Orellana-Paucar et al., 2013; Gawel et al., 2020c, 2021; Gong et al., 2020; Zhang et al., 2020).

Recently, we have validated the method of neurotransmitter analysis (GABA, glutamate, dopamine, and serotonin), but one pooled sample used for determination purposes consisted of 100 larvae (Gawel et al., 2021). When we collected samples consisting of smaller numbers of larvae, the neurotransmitter levels were below the detection limit. Leclercq et al. (2015) analyzed the neurotransmitter levels in larval zebrafish with 6 heads collected per sample, but GABA and glutamate were the only parameters analyzed in this study. Recently, a few more groups have analyzed neurotransmitters and neurochemicals levels (Tufi et al., 2016; Heylen et al., 2021; Jeong et al., 2022), but these proved methodologically different from the above-mentioned papers. In summary, the neurotransmitter analysis method applied in larval zebrafish studies should be further validated.

Other methods that have been described in literature include the transient knockdown of gene encoding targets of the compound of interest, e.g., *stx1b* (Zheng et al., 2018) and *gsk-3ß* (Aourz et al., 2019). The recruitment of macrophages and neutrophils in the *MPO:GFP* line zebrafish (the green fluorescent protein expressed in neutrophils driven by the myeloperoxidase promoter) after exposure to PTZ was also described (Zhang et al., 2020), but there have been few reports on this topic so far.

**Figure 2:**
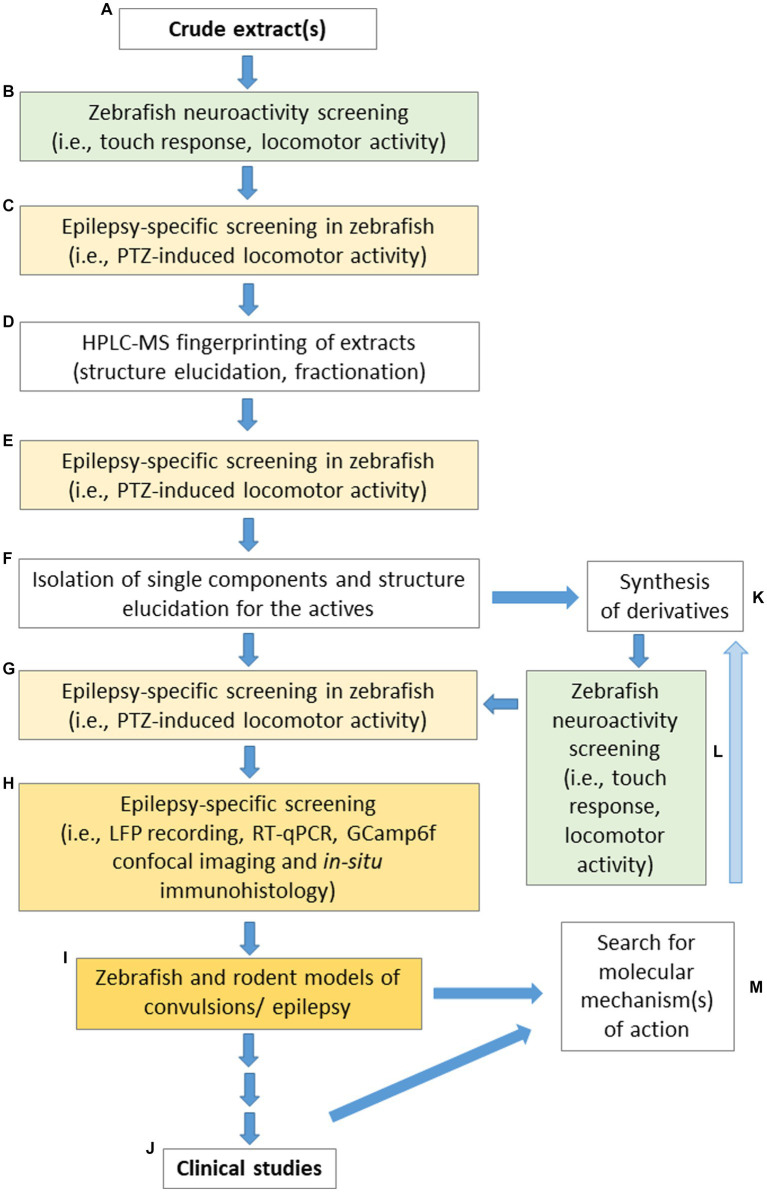
“From tank to bedside and back again in epilepsy”—the suggested pipeline for the search of active components extracted from plant material suitable for the application in anticonvulsant/antiepileptic therapies. The following steps are recommended: **(A)** optimized extraction protocol; **(B)** initial neuroactivity screening; **(C)** epilepsy-specific screening in the PTZ-induced seizure assay performed on zebrafish; **(D)** fingerprinting of extracts by HPLC-MS and the extract fractionation by modern chromatographic techniques (e.g., Flash/CPC/CPE/prep-HPLC/CE/MPLC/prep-TLC) to obtain active fractions or single isolates; **(E)** examination of the fractions and isolates in the PTZ-induced seizure assay; **(F)** bio guided fractionation to obtain single molecules or small groups of compounds; **(G)** further biological activity screening in the PTZ-induced seizure assay; **(H)** conduction of other experiments (e.g., LFP recordings or *in situ* hybridization) to confirm the promising behavioral observations; **(I)** testing of selected promising metabolites in a range of zebrafish and rodent models of convulsions to verify their activity; **(J)** referral of drug candidates for clinical trials; **(K)** synthesis of derivatives; **(L)** initial neuroactivity screening of newly synthesized derivatives; **(M)** use of data obtained from preclinical and clinical studies to search FIGURE 2 (Continued)for the molecular mechanism of action which will be utilized to synthesize new improved derivatives **(K)**, screening these for their anticonvulsant activity **(L,G–J,M)**. The steps are highlighted in colors: the green background indicates the first set of behavioral tests that assess the effect of the tested compounds on the neuroactive performance of zebrafish; the yellow background indicates a set of tests oriented to study anticonvulsant effects; more intense color indicates more sophisticated procedures. LFP, local field potential; PTZ, pentylenetetrazole; RT-qPCR, quantitative reverse transcription PCR.

In relation to all the above-mentioned limitations and challenges, Figure 2 presents a proposed pipeline for different laboratories specializing in studies devoted to screening plant-derived drugs with potential anticonvulsant properties using zebrafish, to make the obtained data both comparable and repeatable.

## 5. Conclusion

This article contains a review of recent findings and challenges linked to searching for plant-derived drugs with anticonvulsant potency in zebrafish-based screening assays. The conducted analysis confirms the usefulness of *Danio rerio* models (both PTZ and genetic) in epilepsy research. The example of berberine shows that the zebrafish model revealed its anticonvulsant effect better than the PTZ convulsive model in rodents, in which it proved ineffective. In our opinion, zebrafish as a screening tool has a chance to bring a breakthrough in identifying new drug leads of plant origin, which might be used as new anticonvulsants. However, more work is advisable to implement new models of seizures/epilepsy, as well to validate the techniques/conditions used in different laboratories. The next step, therefore, should be to study the mechanisms of the anticonvulsant activity of the hit molecules, as otherwise those substances have a lower chance of entering clinical trials.

## Author contributions

KG: conceptualization and supervision. BK, DN, UK, and KG: writing of original draft. DN, UK, WK-K, WAT, and KG: review and editing. DN, KK-T, and WAT: tables. DN, UK, and KG: figures. All authors contributed to the article and approved the submitted version.

## Funding

This paper was partially supported by the DS 448 from Medical University of Lublin, Poland and by National Science Center, Poland within OPUS grant (project no 2021/41/B/NZ4/00337).

## Conflict of interest

The authors declare that the research was conducted in the absence of any commercial or financial relationships that could be construed as a potential conflict of interest.

## Publisher’s note

All claims expressed in this article are solely those of the authors and do not necessarily represent those of their affiliated organizations, or those of the publisher, the editors and the reviewers. Any product that may be evaluated in this article, or claim that may be made by its manufacturer, is not guaranteed or endorsed by the publisher.
