# Supplementary material for: Zebrafish as a robust preclinical platform for screening plant-derived drugs with anticonvulsant properties—a review
Source: Front Mol Neurosci. 2023 Aug 28;16:1221665. doi: 10.3389/fnmol.2023.1221665 (PMC10493295; doi:10.3389/fnmol.2023.1221665)
Supplement: Supplementary file 1 [file Table_1.docx]

**Supplementary Table 1**. Summary of anticonvulsant effect(s) of plant extracts and purified drugs isolated from plant material in different rodent models of seizures/epilepsy.

| **Species** | **Drug/**  **extract studied**  (active dose, route of administration, pretreatment time) | **Model**  **of seizures/epilepsy** | **EEG recording** | **Behavioral measure**  **of convulsions** | **Other findings** | **Suggested mechanism of anticonvulsant action** | **Ref.** |
| --- | --- | --- | --- | --- | --- | --- | --- |
| *Berberis sp.* | berberine  (20 mg/kg; ip;  30 min) | PTZ-induced seizure test in mice | – | ***NS*** latency and duration of myoclonic jerks  ***NS*** occurrence of seizure  ***NS***  mortality | – | – | (Bhutada et al., 2010) |
|  |  | MES test in mice | – | **** HLTE duration  **** HLTE occurrence  **** mortality | – | – |  |
|  |  | KA-induced seizure test in mice | – | **** latency to tonic-clonic seizures  **** mortality | – | – |  |
|  | berberine  (5 mg/kg; ip;  30 min) | PTZ-induced seizure test in mice | – | ***NS*** seizure occurrence |  |  | (Shanbhag et al., 1970) |
|  |  | MES test in mice | – | ***NS*** seizure occurrence | – | – |  |
|  | berberine  (400 mg/kg; ip; 30 min) | PTZ-induced seizure test in rats | – | ***NS*** MCS occurrence  ****latency to MCS  ***NS*** GTCS occurrence  ****latency to GTCS  ***NS*** mortality | – | – | (Sadeghnia et al., 2011) |
|  | berberine  (50-200 mg/kg; ip; 40 min) | 4-AP-induced seizure model in rats | – | **** latency to the generalized seizures | **** aspartate release from hippocampus  **** glutamate release from hippocampus | due to reduction of the excitatory (aspartate and glutamate) neurotransmission | (Sadeghnia et al., 2017) |
|  | berberine  (50-100 mg/kg;  ip; daily, for 1 week before KA-injection) | KA-induced temporal lobe epilepsy in rats | – | **** seizure severity within 24 h  **** number of spontaneous seizures after 2 weeks | **** hippocampal nitrite level  **** hippocampal MDA level | at least partially due to the antioxidant activity | (Mojarad and Roghani, 2014) |
|  | berberine  (25-50 mg/kg; po; daily; 1 week before KA administration and 1 week after KA administration) | KA-induced temporal lobe epilepsy in rats | – | **** incidence rate of status epilepticus during first 24 h after KA injection  **** occurrence of spontaneous recurrent seizures at 6^th^ week after KA tretment | **** reactive oxygen species, caspase 3 in hippocampus  **** GSH, Nrf2, heme oygenase 1 level, catalase activityin hippocampus  **** NFκB, toll-like receptor 4, TNF-α, IL-1b in hippocampus  **** degeneration and neuronal loss in CA3 region of hippocampus  **** mossy fiber sprouting in CA3 region of hippocampus | due to suppression of oxidative stress, neuroinflammation and apoptosis processess | (Sedaghat et al., 2017) |
|  | berberine  (25-100 mg/kg; ig; once daily, 7 days before PILO administration) | PILO-induced temporal lobe epilepsy in rats | – | **** latency to the first seizure  **** time to SE  **** percentage of SE  **** mortality | ***NS*** superoxide dismutase activity in hippocampus  **** catalase, glutathione level in hippocampus  **** lipid peroxidation in hippocampus  **** degeneration of neurons in CA1 region of hippocampus  **** memory impairments 2 weeks after pilocarpine-induced SE | due to antioxidant properties | (Gao et al., 2014) |
|  | methanolic extract from *B. integerrima* roots  (140-200 mg/kg; ip; 30 min) | PTZ-induced seizure test in mice | – | **** HLTE latency  ***NS*** mortality after 30 min and 24 h | – | – | (Hosseinzadeh et al., 2013) |
|  | methanolic extract from *B. integerrima* roots  (200 mg/kg; ip; 30 min) | MES test in mice | – | **** HLTE occurrence  ***NS*** HLTE duration  ***NS*** mortality after 30 min and 24 h | – | – |  |
|  | chloroform fraction of methanolic extract from *B. integerrima* roots  (200 mg/kg; ip; 30 min) | PTZ-induced seizure test in mice | – | **** HLTE latency  ***NS*** mortality after 30 min and 24 h | – | – |  |
|  |  | MES test in mice | – | ***NS*** HLTE occurrence  ***NS*** HLTE duration  ***NS*** mortality after 30 min and 24 h | – | – |  |
|  | hydromethanolic fraction of methanolic extract from *B. integerrima* roots  (200 mg/kg; ip; 30 min) | PTZ-induced seizure test in mice | – | **** HLTE latency  **** mortality after 30 min  ***NS*** mortality after 24 h | – | – |  |
|  |  | MES test in mice | – | ***NS*** HLTE occurrence  ***NS*** HLTE duration  ***NS*** mortality after 30 min and 24 h | – | – |  |
|  | hydroalcoholic extract from *B. vulgaris* (400 mg/kg; ip; 30 min) | PTZ-induced seizure test in rats | – |  latency to the onset of seizures   rate of mortality** ** | – | – | (Khosravi Dehaghi et al., 2017) |
|  | methanol fraction of hydroalcoholic extract from *B. vulgaris* (200 mg/kg; ip; 30 min) |  | – |  latency to the onset of seizures   rate of mortality** ** | – | – |  |
| *Cannabis sativa* | CBD  (ip; 60 min) | MES test in mice | – | ED_50_ = 80 mg/kg  (95% CI = 65.5‐96.0) | – | – | (Patra et al., 2019) |
|  | CBD  (ip; 2 h) | MES test in mice | – | ED_50_ = 83.5 mg/kg  (95% CI = 68‐101) | – |  | (Klein et al., 2017) |
|  | CBD  (20-200 ng/mouse; icv; 10 min) | MES test in mice | – | ** **seizure occurrence | - paxilline (potassium BK channel blocker) does not affect anticonvulsant action | – | (Shirazi-zand et al., 2013) |
|  | CBD  (50-100 mg/kg; ip; 60 min) | MEST test in mice | – | ** **seizure threshold | – | partially due to interaction with TRPV1 receptors | (Gray and Whalley, 2020) |
|  | CBD  (100 mg/kg; ip; 60 min) | MEST test in TRPV1 knockout mice | – | ** **seizure threshold | – |  |  |
|  | CBD  (ip; 60 min) | sc PTZ test in mice | – | ED_50_ = 120 mg/kg  (95% CI = 98.5‐146) | – | – | (Patra et al., 2019) |
|  | CBD  (ip; 2 h) | sc PTZ test in mice | – | ED_50_ = 159 mg/kg  (95% CI = 102‐225) |  |  | (Klein et al., 2017) |
|  | CBD  (60 mg/kg; ip; 30 min) | sc PTZ test in mice | – | **** seizure latency  **** seizure duration |  |  | (Vilela et al., 2017) |
|  | CBD  (60 mg/kg; ip; 30 min) | ip PTZ test in mice | **** latency to first seizure  **** total seizure duration | **** seizure latency  **** seizure duration | **** IL-6 level in PFC vs. vehicle+PTZ-treated group  ***NS*** IL-6 level in PFC vs. vehicle+PTZ-treated group  ***NS*** IL-2,IL-4, IL-10, IL-17, TNF-α, IFN-γ level in PFC and HIP vs. vehicle+PTZ-treated group   - CB1, CB2 and TRPV1 receptor antagonists (i.e., AM251, AM630 and SB366771, respectively) reversed anticonvulsant effect | Due to indirect  CB1 and CB2 receptor facilitation and TRPV1 channel desensitization |  |
|  | CBD  (60 mg/kg; ip; 30 min) | iv PTZ test in mice | – | **** threshold for the forelimb clonus |  |  |  |
|  | CBD  (200 ng/mouse; icv; 10 min) | iv PTZ test in mice | – | ***NS*** myoclonic and clonic seizure threshold  ** **tonic seizure threshold | Co-administration of paxilline (potassium BK channel blocker) and CBD attenuated its anticonvulsant effect | part due to the decrease in intracellular Ca levels that is likely mediated by BK channels | (Shirazi-zand et al., 2013) |
|  | CBD  (60 mg/kg; ip; every other day; 30 min before each PTZ injection) | PTZ-induced kindling in mice | – | ** **kindling progression | – | – | (Vilela et al., 2017) |
|  | CBD  (ip; 60 min) | 6 Hz test (32 mA) in mice | – | ED_50_ = 144 mg/kg  (95% CI = 102‐194) | – | – | (Patra et al., 2019) |
|  | CBD  (ip; 60 min) | 6 Hz test (44 mA) in mice | – | ED_50_ = 173 mg/kg  (95% CI = 136‐213) | – | – |  |
|  | CBD  (ip; 2 h) | 6 Hz test (44 mA) in mice | – | ED_50_ = 164 mg/kg  (95% CI = 124‐200) | – | – | (Klein et al., 2017) |
|  | CBD  (ip; 60 min) | Corneal  kindled mice | – | ED_50_ = 115 mg/kg  (95% CI = 77.5‐169) | – | – | (Patra et al., 2019) |
|  | CBD  (ip; 2 h) | Corneal  kindled mice | – | ED_50_ = 119 mg/kg  (95% CI = 89‐150) |  |  | (Klein et al., 2017) |
|  | CBD  (ip; 60 min) | MES test in rats | – | ED_50_ = 53.2 mg/kg  (95% CI = 39.1‐67) | – | – | (Patra et al., 2019) |
|  | CBD  (ip; 2 h) | MES test in rats | – | ED_50_ = 88.9 mg/kg  (95% CI = 69‐124) | – | – | (Klein et al., 2017) |
|  | CBD  (10 mg/kg; iv; 60 min) | PILO‐induced status epilepticus rat model | – | **** maximum seizure severity | – | – | (Patra et al., 2019) |
|  | CBD  (200 mg/kg; po; 8 weeks) | RISE‐SRS model of TLE in rats | – | **** seizure burden ratio | **** motor comorbidities  **** reference memory and working memory | – |  |
|  | CBD  (10-30 mg/kg; ip; 15 min) | Cocaine-induced seizure test in mice | – | **** seizure latency  **** seizure duration | – | – | (Vilela et al., 2015) |
|  | CBD  (15-90 mg/kg; ip; 30 min) | Cocaine-induced seizure test in mice | – | **** seizure duration  **** seizure latency (only 30 mg/kg) | **** glutamate release in hippocampal synaptosomes   - neither CB_1_ receptor antagonist (i.e., AM251) nor the CB_2_ receptor antagonist (i.e., AM630) revert anticonvulsant effect; - mTOR inhibitor (i.e., rapamycin) reversed anticonvulsant effect | due to activation of mTOR with subsequent reduction in glutamate release | (Gobira et al., 2015) |
|  | CBD  (100-200 mg/kg; ip; 60 min) | Scn1a^+/-^ mice  (Dravet syndrome model) | – | **** seizure duration and severity  **** spontaneous seizures frequency | **** autistic-like social deficits  **** GABA_A_ receptor-mediated inhibition  **** excitation/inhibition ratio  **** action potential firing of excitatory neurons | due to the inhibition of the lipid-activated G protein-coupled receptor GPR55 | (Kaplan et al., 2017) |
|  | CBD  (100-300 mg/kg; ip; 2h) | Lamotrigine-resistant amygdala kindled rat | – | no effect | – | – | (Klein et al., 2017) |
|  | CBD (25/mg/kg; po; 60 min) | repeated 6 Hz corneal stimulation test | ***NS*** duration of the ictal ECoG recordings  ***NS*** changes in power band spectrum | ***NS*** GTCS occurrence | ***NS*** FosB/∆FosB immunoreactivity in the l CA1 region of HIP and in the subiculum | – | (Costa et al., 2021) |
|  | CBN  (150 mg/kg; po; 30 min) | iv PTZ test in mice | – | ***NS*** threshold for clonic seizures | – | – | (Chesher and Jackson, 1974) |
|  | CBN  (50-200 mg/kg; po; 60, 120, 180, 240 min) | MES test in mice | – | ***NS*** HLTE duration | – | – |  |
|  | LN;  (350 mg/kg; ip; 30 min) | sc NMDA-induced seizures in mice | – | **** clonic seizure latency  ***NS*** clonic seizure occurrence | – | – | (Elisabetsky et al., 1999) |
|  | LN  (15-30 mM; icv; 5 min) | Quinolinic acid-induced seizures in mice | – | **** clonic seizure occurrence | – | – |  |
|  | LN  (2.2-2.5 g/kg; po; 30 min; 6 injections every third day) | PTZ-induced kindling in mice | – | **** % of convulsions > 3 s  **** kindling progression | ***NS*** L-[^3^H]glutamate binding to cortex membranes | – |  |
|  | LN (ip; 30 min) | sc PTZ test in mice | – | ***NS*** clonic seizure occurrence | ****anticonvulsant effect of diazepam and valproic acid | – | (Elisabetsky and Brum, 2003) |
|  |  | MES test in mice | – | ***NS*** HLTE occurrence | ****anticonvulsant effect of phenytoin and valproic acid | – |  |
|  | THC;  (1-80 mg/kg; po; 30 min) | iv PTZ test in mice | – | ***NS*** threshold for clonic seizures | – | – | (Chesher and Jackson, 1974) |
|  | THC;  (5-10 mg/kg; ip; 15 min) | Cocaine-induced seizure test in mice | – | **** seizure latency  **** seizure duration | - CB1 and CB2 antagonists (i.e., AM251 and AM630, respectively) did not abolish anticonvulsant effect - Combination of CB1 and CB2 antagonists at high doses attenuated anticonvulsant effect | Restoration of glycine receptor dysfunction | (Zou et al., 2020) |
|  | THC  (0.1-0.3 mg/kg; ip; 30 min) | hypertherrmia-induced acute seizures in Scn1a^+/-^ mice (Dravet syndrome model) | – | **** threshold temperature for GTCS | – | – | (Anderson et al., 2020) |
|  | THC  (10-28.5 mg/kg/day; sub-chronic supplementation in chow) | hypertherrmia-induced spontaneous seizures in Scn1a^+/-^ mice (Dravet syndrome model) | – | ***NS*** occurrence of spontaneoushypertherrmia-induced GTCS  ***NS*** frequency of spontaneoushypertherrmia-induced GTCS  ***NS*** mortality | – | – |  |
|  | CBD + THC  (0.1 mg/kg + 12 mg/kg; ip; 30 min) | hypertherrmia-induced acute seizures in Scn1a^+/-^ mice (Dravet syndrome model) | – | **** threshold temperature for GTCS | – | – |  |
|  | CBD + THC  (500 mg/kg/day + 10 mg/kg/day; sub-chronic supplementation in chow) | hypertherrmia-induced spontaneous seizures in Scn1a^+/-^ mice (Dravet syndrome model) | – | ***NS*** spontaneoushypertherrmia-induced GTCS occurrence  ***NS*** spontaneoushypertherrmia-induced GTCS frequency  **** seizure severity and mortality | – | – |  |
|  | CBD + THC  (50 mg/kg + 50 mg/kg; po; 120 min) | MES test in mice | – | ***NS*** HLTE duration | – | – | (Chesher and Jackson, 1974) |
|  | CBD + CBN  (50 mg/kg + 50 mg/kg; po; 120 min) | MES test in mice | – | ***NS*** HLTE duration | – | – |  |
|  | CBN + THC  (50 mg/kg + 50 mg/kg; po; 120 min) | MES test in mice | – | ***NS*** HLTE duration | – | – |  |
|  | CBN + CBD + THC  (50 mg/kg + 50 mg/kg + 50 mg/kg; po; 120 min) | MES test in mice | – | ****HLTE duration | – | – |  |
|  | *C. sativa* oil without volatile components  (at CBD dose of 25/mg/kg; po; 60 min) | Repeated 6 Hz corneal stimulation test | ***NS*** duration of the ictal ECoG recordings  ***NS*** changes in power band spectrum | **** GTCS occurrence | ***NS*** FosB/∆FosB immunoreactivity in the l CA1 region of HIP and in the subiculum | – | (Costa et al., 2021) |
|  | *C. sativa* oil with volatile components;  (at CBD dose of 25/mg/kg; po; 60 min) | Repeated 6 Hz corneal stimulation test | ***NS*** duration of the ictal ECoG recordings  ** **power of delta rhythm  ** **power of theta rhythm | **** GTCS occurrence | ***NS*** FosB/∆FosB immunoreactivity in the l CA1 region of HIP  **** FosB/∆FosB immunoreactivity in the subiculum | – |  |
| *Curcuma longa* | curcumin  (100-200 mg/kg; po; 3 days prior to PILO administration) | Lithium-PILO-induced SE in rats | – | ** **seizure latency  ** **seizure occurrence  ** **SE latency  ** **mortality | ** **SE-iduced cognitive dysunctions  ** **lipid peroxidation content in HIP and straitum  ** **GSH level in HIP and striatum | – | (Ahmad, 2013) |
|  | curcumin  (50-200 mg/kg; po; 60 min) | PTZ-induced kindling in mice | – | ** **kindling progression | ** **malenodialdehyde level in brain  ** **glutatione level in brain | – | (Agarwal et al., 2011) |
|  | curcumin  (80 mg/kg; po; 45 min) | iv PTZ test in mice | – | ***NS*** threshold for myoclonic and generalized clonic seizures  ** **threshold for tonic seizures | - Anticonvulsant effect prevented by revented by non-selective adenosine receptor antagonist (i.e., 8-phenyltheophylline) and adenosine A1 receptor antagonist (i.e., 8-cyclopentyl-1,3-dipropylxanthine) but not by adenosine A 2A receptor antagonist (i.e., -(3-cholorostryl)caffeine); - Anticonvulsant action potentiated by non-selective A 1 /A 2 receptor agonist (i.e, 5′-N-ethylcarboxamidoadenosine) and adenosine A1 receptor agonist (N^6^ –cyclohexyladenosine) but not by adenosine A2A receptor agonist (i.e.,5′-(N-cyclopropyl) carboxamidoadenosine) | direct or indirect activation of adenosine A1 receptor | (Akula and Kulkarni, 2014) |
|  | curcumin  (150 mg/kg; ip; 25 min) | iv PTZ test in mice | – | **** seizure latency  ** **latency to tonic-clonic seizure  ** **duration of tonic and tonic-clonic seizures** **  ***NS*** mortality, falling* * | ** **HTR7 receptor mRNA expression   - 5-HT1A, 5-HT2C and 5-HT4 antagonists diminished anticonvulsant effect - 5-HT7 antagonist strengthen anticonvulsant effect | By increasing the serotonin levels in the brain that influence receptors, including 5-HT1A, 5-HT2C, and 5-HT4 and  likely through the reduction of 5-HT7 gene expression | (Arbabi Jahan et al., 2018) |
|  | curcumin  (300 mg/kg; po; 60 min) | *ip* PTZ test in rats | – | ** **myoclonic jerk latency  50% protection againstGTCS occurrence | **** MDA level in the brain  ** **GSH level in brain | – | (Reeta et al., 2011) |
|  | curcumin  (300 mg/kg; po; 60 min) | MES test in rats | – | 33% protection against HLTE | **** MDA level in the brain  ** **GSH level in brain   - anti-convulsant activity of sub-therapeutic doses of valproate, phenytoin, phenobarbitone and carbamazepine in rats | – | (Reeta et al., 2011) |
|  | α,β-turmerone  (50 mg/kg; iv; 10 min) | iv PTZ test in mice | – | ** **threshold for ear twitch, myoclonic twitch, tail twitch  ** **threshold for forelimb clonus  ** **threshold for falling  ** **threshold for HLTE  ** **threshold for death | – | – | (Orellana-Paucar et al., 2012) |
|  | ar-turmerone (100 mg/kg; iv; 10 min) | iv PTZ test in mice | – | ** **threshold for ear twitch, myoclonic twitch, tail twitch  ** **threshold for forelimb clonus  ** **threshold for falling  ** **threshold for death | – | – |  |
|  | ar-turmerone  (1 and 20 mg/kg; iv; 10 min) | iv PTZ test in mice | – | ** **threshold for HLTE  (only 1 mg/kg)  ** **threshold for death | ***NS*** motor function/balance | – | (Orellana-Paucar et al., 2013) |
|  | ar-turmerone  (0.1-50 mg/kg; ip; 30 min) | 6 Hz psychomotor seizure test in mice | – | **** seizure occurrence** ** | – | – |  |
|  | ar-turmerone (50 mg/kg; ip; 24 h) |  | – | **** seizure occurrence | – | – |  |
|  | aqueous extract of *Curcuma longa*;  (ip; at 14-16 postnatal day) | ip PTZ test in mice | – | ** **latency to first seizure | – | – | (Sharma and Rauniar, 2016) |
|  | aqueous extract of *Curcuma longa*;  (200 mg/kg; po; daily by 21 days) | MES test in mice | – | ** **duration of HLTE | – | – |  |
|  | *Curcuma longa* oil  (50-100 mg/kg; iv; 10 min) | iv PTZ test in mice | – | ** **threshold for ear twitch, myoclonic twitch, tail twitch  ** **threshold for forelimb clonus  ** **threshold for falling (only 100 mg/kg)  ** **threshold for HLTE (only 100 mg/kg)  ** **threshold for death | – | – | (Orellana-Paucar et al., 2012) |
| *Indigofera arrecta* | methanol extract from aerial part of *I. arrecta* (250-1000 mg/kg; ip; 30 min) | sc PTZ test in mice | – | ** **seizure latency  ***NS*** seizure occurrence  ***NS*** mortality | – | – | (Adejoke et al., 2020) |
|  | Indirubin (2-10 mg/kg; ip; 30 min) | 6 Hz-induced seizure test | _ | **** number of mice protected | – | glycogen synthase kinase (GSK)-3 inhibition | (Aourz et al., 2019) |
|  | Indirubin (2-10 mg/kg; ip; 30 min) | iv PTZ test in mice | – | ***NS*** seizure threshold for myoclonic twitch, forelimb clonus, falling, tonic hindlimb extension and death | ­– |  |  |
|  | Indirubin (2-10 mg/kg, ip; 30 min) | PILO-induced seizures in rats | ­– | ** **seizure severity | ­– |  |  |
| *Magnolia officinalis* | magnolol  (40-80 mg/kg; ip; 30 min) | ip PTZ test in mice | **** seizure latency  **** total number of seizure spikes | **** myoclonic jerk latency  **** generalized clonus latency  ** **seizure severity | **** *c-fos* expression in the HIP and priform cortexx   - selective GABAA/benzodiazepine antagonist (i.e. flumazenil) abolished anticonvulsant effect | mediated by the GABA_A_/benzodiazepine receptor complex | (Chen et al., 2011) |
|  | honokiol  (1-5 mg/kg; ip; 30 min) | NMDA-induced seizures in mice | – | **** threshold for rrunning and bouncing clonic seizures  **** HLTE threshold (5mg/kg) | – | – | (Lin et al., 2005) |
|  | honokiol  (1mg/kg/day; po; for 2 constitutive days before the seizure test) | NMDA-induced seizures in mice | – | **** generalized seizure occurrence  **** generalized seizure latency  ** **seizure score | ** **NMDA-induced motor impairment  **** reactive oxigen species formulation in the brain structures  **** synaptosomal Na, K-ATPase nad Mg^2+^-ATPace activity | – | (Chang-Mu et al., 2010) |
|  | *Magnolia officinalis extract* (as vegetable capsules; 300 mg/kg; po; every 24 h for 10 days after the last KA treatment) | KA-induced recurrent SE in immature rats. | **** afterdischarge threshold  **** relative power of delta, theta, alpha, gamma bands  **** relative power of beta band | **** seizure intensity | **** NeunN-immunoreactive neurons in the dorsal HIP | – | (Vega-García et al., 2019) |
|  | ethanol extract of the Magnolia dealbata leaves(300 mg/kg; ip; 30 min) | ip PTZ test in mice | – | ***NS***myoclonus latency  **** clonus latency  **** tonus latency | – | – | (Martínez et al., 2006) |
|  | ethanol extract of the Magnolia dealbata leaves (300 mg/kg; po; 30 min) |  | – | ****myoclonus latency  **** clonus latency  **** tonus latency | – | – |  |
| *Moringa oleifera* | *Moringa oleifera* seed ethanol extract (2 g/kg; ig; 30 min; for 14 days) | ip PTZ test in mice | – | ** **mortality | ** **GABA concentration in HIP  ** **glutamate concentration in HIP  ** **expression of GAD_65_ and α1 subunit off GABA_A_ receptor in HIP  ***NS*** expression of GAD67 and γ2subunit of GABA_A_ receptor | GABAergic system activation | (Liu et al., 2022) |
|  | kaempferol  (2 mg/kg; ig; 30 min; for 14 days) |  | – | ** **mortality |  |  |  |
|  | naringenin  (200 mg/kg; ip; 30 min) | MES test in mice | – | ** **HLTE duration | – | – | (Khodayar et al., 2016) |
|  |  | ip PTZ test in mice | – | ** **seizure latency** **  ** **Straub’s tail latency  ** **myoclonic seizure duration (200 mg/kg) | – | – |  |
|  | naringenin  (100 mg/kg; ip; daily injection for 8 days starting the day before KA injection) | KA-induced seizure model in mice | – | ** **seizure latency | **** granule cell dispersion in the dentate gyrus  **** mammalian target of rapamycin complex 1 (mTORC1) activation in the dentale gyrus  **** TNFα and IL-1β expression in the dentale gyrus | – | (Park et al., 2016) |
|  | naringenin  (20-80 mg/kg; ip; by 7 days before PTZ administration) | ip PTZ test in mice | – | ** **myoclonic jerks latency  ** **GTCS latency (dose 80 mg/kg completely abolished GTCS)  **** GTCS duration | **** PTZ-induced cognitive impairment  **** GSH level in the brain  **** MDA level in the brain  **** TNF-alpha level in the brain | – | (Golechha et al., 2014) |
|  | naringenin (20-40 mg/kg; po; or 15 days) | PILO-induced seizure in mice | – | ** **seizure severity  ** s**eizure latency | **** SOD and CAT activity  **** lipid peroxidation  **** Glutathione reductase level   - neurons morphology improvement in Histologic examination of hippocampal sections |  | (Shakeel et al., 2017) |
|  | NRG-DM  (6.25-25 mg/kg; ip; 30 min) | iv PTZ test in mice | – | ** **tail twitch, falling and HLTE threshold (12.5 mg/kg)  ** **threshold for death | – | – | (Copmans et al., 2018) |
|  |  | 6 Hz test in mice | – | ** **psychomotor seizure occurrence  ** **psychomotor** **seizure duration (6.25 mg/kg) | – | – |  |
|  | pterostilbene  (100-200 mg/kg; ip; 30 min) | iv PTZ test in mice | – | ** **threshold for the first myoclonic twitch  ** **threshold for GTCS  ** **threshold for forelimb tonus | – | – | (Nieoczym et al., 2019) |
|  |  | MEST test in mice | – | ** **HLTE threshold | – | – |  |
|  |  | 6 Hz-induced psychomotor seizure threshold test in mice | – | ** **psychomotor seizure threshold | – | – |  |
|  | pterostilbene  (200 mg/kg; ip; 30 min) | PTZ kindling in mice | – | ** **seizure severity  **** kindling progression | **** GABA concentration in PFC and HIP  ***NS*** glutamate concentration in PFC and HIP |  | (Nieoczym et al., 2021) |
| *Solanum torvum* | methanolic extract from *Solanum torvum* seeds  (10-100 mg/kg; ip; 30 min) | ip PTZ test in mice | – | ** **Straub's tail latency  ** **extensor latency (10-30 mg/kg)  ** **myoclonic jerk latency  ** **clonic convulsion latency  ** **stupor latency (10 and 100 mg/kg) | – | facilitation of GABAergic transmission | (Momin and Mohan, 2011) |
| *Zingiber purpureum* | banglene (50/50 ratio of trans-banglene and cis-banglene) | ip PTZ test in mice | – | ** **seizure score  **** mortality | – | – | (Brillatz et al., 2020) |
| *Zingiber officinale* | hydroethanolic extract from *Zingiber oficinale* roots  (25-100 mg/kg; ip; 2 and 24 h) | iv PTZ test in mice | – | **** first myoclonic seizure threshold  **** generalized clonic seizure threshold (100 mg/kg and 50 mg/kg with 24 h pretreatment)  **** forelimb tonic extension threshold (50 and 100 mg/kg with 2 h pretreatment; 25-100 mg/kg with 24 h pretreatment) | – | – | (Hosseini and Mirazi, 2014, 2015) |
|  | hydroethanolic extract from *Zingiber oficinale* roots  (25-100 mg/kg; ip; daily for 7 days) | iv PTZ test in mice | – | **** first myoclonic seizure threshold  **** generalized clonic seizure threshold (50-100 mg/kg)  **** forelimb tonic extension threshold (50-100 mg/kg) | – | – |  |
|  | essential oil of *Z. officinale*  (50-100 mg/kg; ip; daily for 7 days; 30 min) | PILO-induced seizures in mice | – | ** **seizure latency  ***NS*** mortality | – | – | (Felipe et al., 2008) |
|  | hydroalcoholic extract of *Z. officinale*  (100 mg/kg; ip; daily for 10 days before kindling procedure onset and then administration 60 min before each PTZ injection) | PTZ-induced kindling in mice | – | ** **seizure severity  ** **myoclonic jerk latency  ** **generalized tonic-clonic seizure latency  ** **generalized tonic-clonic seizure duration | ** **NeuN expressing neurons (i.e., mature neurons) in CA3 region of HIP  ** **astrocyte activation in CA3 region of HIP | – | (Naeimi et al., 2018) |

4-AP – 4-aminopyridine; CBD – cannabidiol; CBDVA ­– cannabidivarinic acid; CBGA – cannabigerolic acid; CBGVA – cannabigerovarinic
acid; CBN – cannabinol; CI – confidence interval; GSH – glutathione; GTCS – generalized tonic-clonic seizures; HIP – hippocampus; HLTE – hindlimb tonic extension; ip – intraperitoneally; iv – intravenous; KA – kainic acid; KFD – kaempferide (4'-*O*-methyl kaempferol); KFL – kaempferol; LFP – local field potential; LN – linalool; MCS – minimal clonic seizures; MDA – malondialdehyde; MES – maximal electroshock; MEST – maximal electroshock threshold test; MNDA – NRG – naringenin; NRG-DM – naringenin 4',7-dimethyl ether; NRG-M – naringenin 7-*O*-methyl ether; *NS* – not statistically significant; PFC – prefrontal cortex; PILO – pilocarpine; po – *per os*;PTZ – pentetrazole; RISE-SRS – reduced intensity status epilepticus–spontaneous recurrent seizures; SE – status epilepticus; THC – *delta*(9)-tetrahydrocannabinol

**References**

Adejoke, A., Halima, H., Aduku, O., and Dauda, A. (2020). Isolation and Anticonvulsant Studies of Methanol extract of the aerial part of Indigofera arrecta Hochst. Ex. A. Rich (Papilioaceae). Nigerian Journal of Pharmaceutical Research *19*, 94-103.

Agarwal, N.B., Jain, S., Agarwal, N.K., Mediratta, P.K., and Sharma, K.K. (2011). Modulation of pentylenetetrazole-induced kindling and oxidative stress by curcumin in mice. Phytomedicine *18*, 756-759.

Ahmad, M. (2013). Protective effects of curcumin against lithium-pilocarpine induced status epilepticus, cognitive dysfunction and oxidative stress in young rats. Saudi J Biol Sci *20*, 155-162.

Akula, K.K., and Kulkarni, S.K. (2014). Effect of curcumin against pentylenetetrazol-induced seizure threshold in mice: possible involvement of adenosine A1 receptors. Phytother Res *28*, 714-721.

Anderson, L.L., Low, I.K., McGregor, I.S., and Arnold, J.C. (2020). Interactions between cannabidiol and Δ(9) -tetrahydrocannabinol in modulating seizure susceptibility and survival in a mouse model of Dravet syndrome. Br J Pharmacol *177*, 4261-4274.

Aourz, N., Serruys, A.-S.K., Chabwine, J.N., Balegamire, P.B., Afrikanova, T., Edrada-Ebel, R., Grey, A.I., Kamuhabwa, A.R., Walrave, L., Esguerra, C.V.*, et al.* (2019). Identification of GSK-3 as a Potential Therapeutic Entry Point for Epilepsy. ACS Chemical Neuroscience *10*, 1992-2003.

Arbabi Jahan, A., Rad, A., Ghanbarabadi, M., Amin, B., and Mohammad-Zadeh, M. (2018). The role of serotonin and its receptors on the anticonvulsant effect of curcumin in pentylenetetrazol-induced seizures. Life Sci *211*, 252-260.

Bhutada, P., Mundhada, Y., Bansod, K., Dixit, P., Umathe, S., and Mundhada, D. (2010). Anticonvulsant activity of berberine, an isoquinoline alkaloid in mice. Epilepsy Behav *18*, 207-210.

Brillatz, T., Kubo, M., Takahashi, S., Jozukuri, N., Takechi, K., Queiroz, E.F., Marcourt, L., Allard, P.M., Fish, R., Harada, K.*, et al.* (2020). Metabolite Profiling of Javanese Ginger Zingiber purpureum and Identification of Antiseizure Metabolites via a Low-Cost Open-Source Zebrafish Bioassay-Guided Isolation. J Agric Food Chem *68*, 7904-7915.

Chang-Mu, C., Jen-Kun, L., Shing-Hwa, L., and Shoei-Yn, L.S. (2010). Characterization of neurotoxic effects of NMDA and the novel neuroprotection by phytopolyphenols in mice. Behav Neurosci *124*, 541-553.

Chen, C.R., Tan, R., Qu, W.M., Wu, Z., Wang, Y., Urade, Y., and Huang, Z.L. (2011). Magnolol, a major bioactive constituent of the bark of Magnolia officinalis, exerts antiepileptic effects via the GABA/benzodiazepine receptor complex in mice. British Journal of Pharmacology *164*, 1534-1546.

Chesher, G.B., and Jackson, D.M. (1974). Anticonvulsant effects of cannabinoids in mice: Drug interactions within cannabinoids and cannabinoid interactions with phenytoin. Psychopharmacologia *37*, 255-264.

Copmans, D., Orellana-Paucar, A.M., Steurs, G., Zhang, Y., Ny, A., Foubert, K., Exarchou, V., Siekierska, A., Kim, Y., De Borggraeve, W.*, et al.* (2018). Methylated flavonoids as anti-seizure agents: Naringenin 4',7-dimethyl ether attenuates epileptic seizures in zebrafish and mouse models. Neurochem Int *112*, 124-133.

Costa, A.M., Senn, L., Anceschi, L., Brighenti, V., Pellati, F., and Biagini, G. (2021). Antiseizure Effects of Fully Characterized Non-Psychoactive Cannabis sativa L. Extracts in the Repeated 6-Hz Corneal Stimulation Test. Pharmaceuticals (Basel) *14*.

Elisabetsky, E., and Brum, L. (2003). Linalool as active component of traditional remedies: anticonvulsant properties and mechanisms of action. Curare *26*, 45-52.

Elisabetsky, E., Brum, L.F., and Souza, D.O. (1999). Anticonvulsant properties of linalool in glutamate-related seizure models. Phytomedicine *6*, 107-113.

Felipe, C.F.B., Fonsecirc, K.S., Barbosa, r.L.d.R., Bezerra, J.N.S., Manoel, Neto, R.A., Franccedil, M.M.d., Fonteles, A.A., and Viana, G.S.B. (2008). Alterations in behavior and memory induced by the essential oil of Zingiber officinale Roscoe (ginger) in mice are cholinergic-dependent. Journal of Medicinal Plants Research *2*, 163-170.

Gao, F., Gao, Y., Liu, Y.F., Wang, L., and Li, Y.J. (2014). Berberine exerts an anticonvulsant effect and ameliorates memory impairment and oxidative stress in a pilocarpine-induced epilepsy model in the rat. Neuropsychiatr Dis Treat *10*, 2139-2145.

Gobira, P.H., Vilela, L.R., Gonçalves, B.D.C., Santos, R.P.M., de Oliveira, A.C., Vieira, L.B., Aguiar, D.C., Crippa, J.A., and Moreira, F.A. (2015). Cannabidiol, a Cannabis sativa constituent, inhibits cocaine-induced seizures in mice: Possible role of the mTOR pathway and reduction in glutamate release. NeuroToxicology *50*, 116-121.

Golechha, M., Sarangal, V., Bhatia, J., Chaudhry, U., Saluja, D., and Arya, D.S. (2014). Naringin ameliorates pentylenetetrazol-induced seizures and associated oxidative stress, inflammation, and cognitive impairment in rats: possible mechanisms of neuroprotection. Epilepsy Behav *41*, 98-102.

Gray, R.A., and Whalley, B.J. (2020). The proposed mechanisms of action of CBD in epilepsy. Epileptic Disord *22*, 10-15.

Hosseini, A., and Mirazi, N. (2014). Acute administration of ginger (Zingiber officinale rhizomes) extract on timed intravenous pentylenetetrazol infusion seizure model in mice. Epilepsy Research *108*, 411-419.

Hosseini, A., and Mirazi, N. (2015). Alteration of pentylenetetrazole-induced seizure threshold by chronic administration of ginger (Zingiber officinale) extract in male mice. Pharmaceutical Biology *53*, 752-757.

Hosseinzadeh, H., Ramezani, M., Shafaei, H., and Taghiabadi, E. (2013). Anticonvulsant Effect of Berberis integerrima L. Root Extracts in Mice. Journal of Acupuncture and Meridian Studies *6*, 12-17.

Kaplan, J.S., Stella, N., Catterall, W.A., and Westenbroek, R.E. (2017). Cannabidiol attenuates seizures and social deficits in a mouse model of Dravet syndrome. Proc Natl Acad Sci U S A *114*, 11229-11234.

Khodayar, M., Salehi, S., Rezaei, M., Siahpoosh, A., Khazaei, A., and Houshmand, G. (2016). Evaluation of the Effect of Naringenin on Pentylenetetrazole and Maximal Electroshock-Induced Convulsions in Mice. Jundishapur Journal of Natural Pharmaceutical Products *Inpress*.

Khosravi Dehaghi, N., Zeraati, M., Kamali, J., Ghorbani Nohooji, M., and Rahimzadeh, M. (2017). Effects of Berberis vulgaris fractions on PTZ Induced seizure in male rats. Research Journal of Pharmacognosy *4*, 101-101.

Klein, B.D., Jacobson, C.A., Metcalf, C.S., Smith, M.D., Wilcox, K.S., Hampson, A.J., and Kehne, J.H. (2017). Evaluation of Cannabidiol in Animal Seizure Models by the Epilepsy Therapy Screening Program (ETSP). Neurochem Res *42*, 1939-1948.

Lin, Y.-R., Chen, H.-H., Ko, C.-H., and Chan, M.-H. (2005). Differential inhibitory effects of honokiol and magnolol on excitatory amino acid-evoked cation signals and NMDA-induced seizures. Neuropharmacology *49*, 542-550.

Liu, W.L., Wu, B.F., Shang, J.H., Wang, X.F., Zhao, Y.L., and Huang, A.X. (2022). Moringa oleifera seed ethanol extract and its active component kaempferol potentiate pentobarbital-induced sleeping behaviours in mice via a GABAergic mechanism. Pharm Biol *60*, 810-824.

Martínez, A.L., Domínguez, F., Orozco, S., Chávez, M., Salgado, H., González, M., and González-Trujano, M.E. (2006). Neuropharmacological effects of an ethanol extract of the Magnolia dealbata Zucc. leaves in mice. Journal of Ethnopharmacology *106*, 250-255.

Mojarad, T.B., and Roghani, M. (2014). The Anticonvulsant and Antioxidant Effects of Berberine in Kainate-induced Temporal Lobe Epilepsy in Rats. Basic Clin Neurosci *5*, 124-130.

Momin, R., and Mohan, M. (2011). Anxiolytic-Like Actions of Methanolic Extract of Solanum Torvum (Solanaceae) Seeds in Mice. Pharmacologyonline *2*.

Naeimi, R., Ghasemi-Kasman, M., Kazemi, S., Ashrafpour, M., Moghadamnia, A.A., and Pourabdolhossein, F. (2018). Zingiber officinale extract pre-treatment ameliorates astrocytes activation and enhances neuroprotection in pentylenetetrazol-induced kindling model of epilepsy in mice. Physiol-Pharmacol *22*, 92-102.

Nieoczym, D., Socała, K., Gawel, K., Esguerra, C.V., Wyska, E., and Wlaź, P. (2019). Anticonvulsant Activity of Pterostilbene in Zebrafish and Mouse Acute Seizure Tests. Neurochem Res *44*, 1043-1055.

Nieoczym, D., Socała, K., Zelek-Molik, A., Pieróg, M., Przejczowska-Pomierny, K., Szafarz, M., Wyska, E., Nalepa, I., and Wlaź, P. (2021). Anticonvulsant effect of pterostilbene and its influence on the anxiety- and depression-like behavior in the pentetrazol-kindled mice: behavioral, biochemical, and molecular studies. Psychopharmacology (Berl) *238*, 3167-3181.

Orellana-Paucar, A.M., Afrikanova, T., Thomas, J., Aibuldinov, Y.K., Dehaen, W., de Witte, P.A., and Esguerra, C.V. (2013). Insights from zebrafish and mouse models on the activity and safety of ar-turmerone as a potential drug candidate for the treatment of epilepsy. PLoS One *8*, e81634.

Orellana-Paucar, A.M., Serruys, A.S., Afrikanova, T., Maes, J., De Borggraeve, W., Alen, J., León-Tamariz, F., Wilches-Arizábala, I.M., Crawford, A.D., de Witte, P.A.*, et al.* (2012). Anticonvulsant activity of bisabolene sesquiterpenoids of Curcuma longa in zebrafish and mouse seizure models. Epilepsy Behav *24*, 14-22.

Park, J., Jeong, K.H., Shin, W.-H., Bae, Y.-S., Jung, U.J., and Kim, S.R. (2016). Naringenin ameliorates kainic acid-induced morphological alterations in the dentate gyrus in a mouse model of temporal lobe epilepsy. NeuroReport *27*.

Patra, P.H., Barker-Haliski, M., White, H.S., Whalley, B.J., Glyn, S., Sandhu, H., Jones, N., Bazelot, M., Williams, C.M., and McNeish, A.J. (2019). Cannabidiol reduces seizures and associated behavioral comorbidities in a range of animal seizure and epilepsy models. Epilepsia *60*, 303-314.

Reeta, K.H., Mehla, J., Pahuja, M., and Gupta, Y.K. (2011). Pharmacokinetic and pharmacodynamic interactions of valproate, phenytoin, phenobarbitone and carbamazepine with curcumin in experimental models of epilepsy in rats. Pharmacology Biochemistry and Behavior *99*, 399-407.

Sadeghnia, H.R., Darbarpanah, S., and Hosseini, S.M. (2011). Effect of berberine on pentylenetetrazol-induced seizures in rats. Avicenna Journal of Phytomedicine *1*, 78-82.

Sadeghnia, H.R., Taji, A.R., Forouzanfar, F., and Hosseinzadeh, H. (2017). Berberine attenuates convulsing behavior and extracellular glutamate and aspartate changes in 4-aminopyridine treated rats. Iran J Basic Med Sci *20*, 588-593.

Sedaghat, R., Taab, Y., Kiasalari, Z., Afshin-Majd, S., Baluchnejadmojarad, T., and Roghani, M. (2017). Berberine ameliorates intrahippocampal kainate-induced status epilepticus and consequent epileptogenic process in the rat: Underlying mechanisms. Biomed Pharmacother *87*, 200-208.

Shakeel, S., Rehman, M., Tabassum, N., Amin, U., and Mir, M. (2017). Effect of Naringenin (A naturally occurring flavanone) Against Pilocarpine-induced Status Epilepticus and Oxidative Stress in Mice. Pharmacognosy Magazine *13*, S154-S160.

Shanbhag, S.M., Kulkarni, H.J., and Gaitonde, B.B. (1970). PHARMACOLOGICAL ACTIONS OF BERBERINE ON THE CENTRAL NERVOUS SYSTEM. Japanese Journal of Pharmacology *20*, 482-487.

Sharma, A., and Rauniar, G.P. (2016). Anticonvulsive Effects of Purified Curcuma Longa in Mice. International Journal of Health Sciences and Research *6*, 236-241.

Shirazi-zand, Z., Ahmad-Molaei, L., Motamedi, F., and Naderi, N. (2013). The role of potassium BK channels in anticonvulsant effect of cannabidiol in pentylenetetrazole and maximal electroshock models of seizure in mice. Epilepsy Behav *28*, 1-7.

Vega-García, A., Santana-Gómez, C.E., Rocha, L., Magdaleno-Madrigal, V.M., Morales-Otal, A., Buzoianu-Anguiano, V., Feria-Romero, I., and Orozco-Suárez, S. (2019). Magnolia officinalis reduces the long-term effects of the status epilepticus induced by kainic acid in immature rats. Brain Research Bulletin *149*, 156-167.

Vilela, L.R., Gomides, L.F., David, B.A., Antunes, M.M., Diniz, A.B., Moreira Fde, A., and Menezes, G.B. (2015). Cannabidiol rescues acute hepatic toxicity and seizure induced by cocaine. Mediators Inflamm *2015*, 523418.

Vilela, L.R., Lima, I.V., Kunsch, É.B., Pinto, H.P.P., de Miranda, A.S., Vieira, É.L.M., de Oliveira, A.C.P., Moraes, M.F.D., Teixeira, A.L., and Moreira, F.A. (2017). Anticonvulsant effect of cannabidiol in the pentylenetetrazole model: Pharmacological mechanisms, electroencephalographic profile, and brain cytokine levels. Epilepsy & Behavior *75*, 29-35.

Zou, G., Zuo, X., Chen, K., Ge, Y., Wang, X., Xu, G., Wang, H., Miao, C., Xu, Z., Tian, S.*, et al.* (2020). Cannabinoids Rescue Cocaine-Induced Seizures by Restoring Brain Glycine Receptor Dysfunction. Cell Reports *30*, 4209-4219.e4207.
